# Comparative Genomic Analysis Uncovered Evolution of Pathogenicity Factors, Horizontal Gene Transfer Events, and Heavy Metal Resistance Traits in Citrus Canker Bacterium *Xanthomonas citri* subsp. *citri*

**DOI:** 10.3389/fmicb.2021.731711

**Published:** 2021-09-07

**Authors:** Chien-Jui Huang, Ting-Li Wu, Po-Xing Zheng, Jheng-Yang Ou, Hui-Fang Ni, Yao-Cheng Lin

**Affiliations:** ^1^Department of Plant Medicine, National Chiayi University, Chiayi, Taiwan; ^2^Biotechnology Center in Southern Taiwan, Agricultural Biotechnology Research Center, Academia Sinica, Tainan, Taiwan; ^3^Department of Plant Protection, Chiayi Agricultural Experiment Station, Taiwan Agricultural Research Institute, Chiayi, Taiwan

**Keywords:** *Xanthomonas citri* subsp. *citri*, copper resistance, whole-genome sequence, TALE, plasmid, plasticity

## Abstract

**Background:** Worldwide citrus production is severely threatened by Asiatic citrus canker which is caused by the proteobacterium *Xanthomonas citri* subsp. *citri*. Foliar sprays of copper-based bactericides are frequently used to control plant bacterial diseases. Despite the sequencing of many *X. citri* strains, the genome diversity and distribution of genes responsible for metal resistance in *X. citri* subsp. *citri* strains from orchards with different management practices in Taiwan are not well understood.

**Results:** The genomes of three *X. citri* subsp. *citri* strains including one copper-resistant strain collected from farms with different management regimes in Taiwan were sequenced by Illumina and Nanopore sequencing and assembled into complete circular chromosomes and plasmids. CRISPR spoligotyping and phylogenomic analysis indicated that the three strains were located in the same phylogenetic lineages and shared ∼3,000 core-genes with published *X. citri* subsp. *citri* strains. These strains differed mainly in the CRISPR repeats and pathogenicity-related plasmid-borne transcription activator-like effector (TALE)-encoding *pthA* genes. The copper-resistant strain has a unique, large copper resistance plasmid due to an unusual ∼40 kbp inverted repeat. Each repeat contains a complete set of the gene cluster responsible for copper and heavy metal resistance. Conversely, the copper sensitive strains carry no metal resistance genes in the plasmid. Through comparative analysis, the origin and evolution of the metal resistance clusters was resolved.

**Conclusion:** Chromosomes remained constant among three strains collected in Taiwan, but plasmids likely played an important role in maintaining pathogenicity and developing bacterial fitness in the field. The evolution of pathogenicity factors and horizontal gene transfer events were observed in the three strains. These data suggest that agricultural management practices could be a potential trigger for the evolution of citrus canker pathogens. The decrease in the number of CRISPR repeats and *pthA* genes might be the result of adaptation to a less stressful environment. The metal resistance genes in the copper resistant *X. citri* strain likely originated from the Mauritian strain not the local copper-resistant *X. euvesicatoria* strain. This study highlights the importance of plasmids as ‘vehicles’ for exchanging genetic elements between plant pathogenic bacteria and contributing to bacterial adaptation to the environment.

## Introduction

*Xanthomonas* spp. are a large group of Gram-negative bacteria that cause disease in more than 400 different plant hosts (Timilsina et al., 2020); however, the host range of the individual species is often restricted to a single or a handful of plants in the same botanical family. To aid explanation of this phenomenon, the term “pathovar” was coined and is defined as an intra-subspecific group of strains causing the same disease with host and tissue specificity (Timilsina et al., 2020). Most *Xanthomonas* species infect plants by first colonizing the surface of aerial organs then entering through stomata or wounds; the host may show symptoms. Asiatic citrus canker caused by *Xanthomonas citri* subsp. *citri* is a serious threat to citrus production in most citrus-growing regions in the world (Brunings and Gabriel, 2003; Ference et al., 2018). *X. citri* subsp. *citri* is a genetically monomorphic bacterium (Leduc et al., 2015; Richard et al., 2017b) and has spread geographically from its Asiatic origin to many citrus-growing regions including Taiwan (Pruvost et al., 2014; Leduc et al., 2015; Huang and Ni, 2017). Recent advances in high throughput sequencing have made it possible to sequence the whole genomes of groups within the microbial community rather than a handful of loci (multilocus sequence typing, MLST) and the reconstruction of the repetitive sequence regions such as the complete CRISPR unit (CRISPR spoligotyping) (Jeong et al., 2019). These advances have allowed the detailed study of the evolution, ecology and dissemination of bacterial pathogens (Vinatzer et al., 2014; Timilsina et al., 2019). Genetically monomorphic bacteria have been considered to have low adaptive potential because of low genetic variability (Achtman, 2008). Yet, how these bacteria adapted to diverse environmental conditions and evolved resistance to antibacterial compounds remains unclear.

Citrus canker pathogens are commonly classified into the three pathotypes: A, B and C. Pathotype A was first reported in Asia in the early nineteenth century (Fawcett and Jenkins, 1933) and later spread to all citrus producing regions worldwide. The first genome of *X. citri* subsp. *citri* strain 306 was completely sequenced in 2002 (da Silva et al., 2002). Two variant forms of pathotype A, namely A^∗^ and A^W^, have been found in Southeast Asia and Southern Florida in the past 30 years (Timilsina et al., 2020). These variants of the pathotype A showed apparent intraspecific diversity and host specialization. After the reference genome of the pathotype A strain 306 was sequenced, a combination of whole genome sequencing and comparative analysis has contributed to the discovery of polymorphisms associated with potential mechanisms of adaptation in genetically monomorphic bacterium *X. citri* subsp. *citri* (Zhang et al., 2015; Richard et al., 2017b; Gochez et al., 2018). To date, multiple factors including transcription activator-like effectors (TALEs), plasmid-mediated horizontal gene transfers and transposons have been found to play important roles in adaptation, evolution and spread of pathogenicity determinants of *X. citri* subsp. *citri* (Ferreira et al., 2015; Richard et al., 2017b, 2021; Gochez et al., 2018).

TALEs belonging to the PthA family of type III secretion system effector proteins (T3SEs) are the main pathogenicity factor of *X. citri* subsp. *citri* (Brunings and Gabriel, 2003; Abe and Benedetti, 2016; Roeschlin et al., 2019). When injected into host cells, PthA proteins activate expression of disease susceptibility or resistance genes (Brunings and Gabriel, 2003; Abe and Benedetti, 2016; Roeschlin et al., 2019). PthA proteins consist of an N-terminal region for secretion, a central DNA-binding domain and a C-terminal region containing nuclear localization signals and an acidic transcriptional activation domain (Boch and Bonas, 2010). The central DNA-binding domain of the PthA family is composed of almost identical tandem repeats of 33 to 34 amino acids (Boch and Bonas, 2010). Each repeat contains a repeat-variable diresidue (RVD) at the 12th and 13th positions (Boch and Bonas, 2010). In addition, the number of tandem repeats are variable among PthA proteins (Brunings and Gabriel, 2003; Abe and Benedetti, 2016; Roeschlin et al., 2019). The reference *X. citri* subsp. *citri* strain 306 carries four TALE-encoding *pthA* genes located on the plasmids pXAC33 (*pthA1* and *pthA2*) and pXAC64 (*pthA3* and *pthA4*) (da Silva et al., 2002). The *pthA1*, *pthA2*, *pthA3* and *pthA4* genes of strain 306 harbor 16.5, 15.5, 15.5, and 17.5 copies of repeats, respectively (da Silva et al., 2002). Recently, comparative analysis of completely sequenced plasmids from *X. citri* subsp. *citri* revealed clues to rearrangements of plasmids and reshuffling of TALEs among citrus canker strains (Gochez et al., 2018). Furthermore, an experimental evolution study showed that in less than 30 cycles of repeated infections, *X. citri* subsp. *citri* could accumulate sufficient mutations and rearrangements of repeats of TALEs to cause pathogenicity in incompatible hosts (Teper and Wang, 2021).

Copper-based bactericides have been widely used for control of plant bacterial diseases throughout the world. However, frequent applications of copper-based bactericides induce the evolution and development of bacterial strains that are either resistant or tolerant to copper (Hseu and Hsu, 1991; Wu et al., 1995; Canteros et al., 2008; Behlau et al., 2012b, 2013). Copper resistant (Cu^R^) strains of *X. citri* subsp. *citri* have been found across the world (Behlau et al., 2011, 2013). Large-sized plasmids carrying copper resistance genes (*cop* genes) are predominantly present in Cu^R^ xanthomonads (Behlau et al., 2011, 2012a). The *cop* genes in xanthomonads associated with citrus and solanaceous hosts have been identified and organized in a cluster (Behlau et al., 2011; Timilsina et al., 2019). In Cu^R^ *Xanthomonas* strains, *copL*, *copA*, and *copB* genes in the *cop* cluster play a major role in copper resistance (Behlau et al., 2011). In addition to the *copLAB* cluster, the plasmid-borne cluster of *copABCD* genes has been also identified in Cu^R^ strains of *X. arboricola* pv. *juglandis* (Lee et al., 1994) and *X. citri* subsp. *citri* (Richard et al., 2017b). Copper resistance plasmids of xanthomonads can be mobilized from a donor cell to a copper sensitive (Cu^S^) recipient cell through conjugation (Behlau et al., 2011, 2012a). Recently, copper tolerant (Cu^T^) *X. ctri* subsp. *citri* strains whose chromosomal genes *cohA* and *cohB* (homologous to *copA* and *copB)* were increasingly expressed in the presence of copper, were found in Brazil, but they are not precursors of Cu^R^ strains (Marin et al., 2019).

Asiatic citrus canker is an important epidemic disease of citrus production worldwide. Previously we used phylogenetic analysis of copper resistance genes *copLAB* in combination with polymorphism analysis of complete *copB* genes to track the possible origin of Cu^R^ *X. citri* subsp. *citri* strains from Taiwan (Lai et al., 2021). However, hitherto nothing has been known about the detailed genome composition of *X. citri* subsp. *citri* in orchards located in different regions managed under distinct agricultural practices. Thus, the aim of this study was to explore the genetic basis of *X. citri* subsp. *citri* by comparative analysis of complete genomes of three strains collected from two regions in Taiwan, including one Cu^R^ strain. The high quality genome assembly and annotation of the three strains were compared with published *X. citri* genomes by CRISPR spoligotyping and core genome analysis to reveal the phylogenetic positions of these three strains. By analyzing the structure and variation of the *pthA* genes, we provided evidence of plasmid fusion in the sequenced genomes. Furthermore, comparative analysis of the copper and arsenate gene cluster helped decipher the origin of citrus canker pathogens with resistance to either copper or heavy metals.

## Materials and Methods

### Collection of Strains

Two *X. citri* subsp. *citri* strains B2 and T4 were isolated from the leaves of *Citrus reticulata* cvs. “Shiranui” and “Tainung Giant,” respectively, from a commercial citrus orchard in Taichung, Taiwan (Geolocation: 24.27 N, 120.78 E), where copper-based bactericides and fungicides have been routinely applied during the citrus growing season, on October 7, 2016. The other strain, SN3-3, was collected from the leaf of *Citrus sinensis* cv. “Suenaga” in an orchard with minimal management that has not applied bactericides and fungicides in Chiayi, Taiwan, (Geolocation: 243.48 N, 120.46 E, Chiayi Agricultural Experiment Branch, Taiwan Agricultural Research Institute) on April 14, 2016.

### Phenotypic Characterization

The pathogenicity of the three strains was tested based on the previously published method (Huang and Ni, 2017). Briefly, leaves of citrus cv. Murcott were infiltrated with bacterial suspensions (1 × 10^6^ CFU/ml). Symptom development was observed 14 days after the inoculation.

Copper sensitivity test was performed according to the method in Lai et al. (2021). The three strains, cultured overnight on NA (Nutrient agar, Difco) plates, were streaked on NA plates supplemented with 0, 0.1, 0.2, 0.4, 0.6, 0.7, 0.8, 1.6, and 3.2 mM CuSO_4_. Stains sensitive, tolerant, or resistant to copper were differentiated by their ability to grow on NA plates with maximum concentrations of ≤0.6, 0.6–0.8 and ≥0.8 mM CuSO_4_, respectively, as rated by Behlau et al. (2013) and Marin et al. (2019).

### Illumina Data Generation

Genomic DNA from the three strains was prepared using Bacteria Genomic DNA kit (Geneaid, Taiwan). Nuclei were isolated according to the manufacturer’s instructions. Purity and quantity of DNA samples were estimated using the Qubit dsDNA HS Assay Kit (Thermo-Fisher Scientific) and Agilent BioAnalyzer 2100 High Sensitivity DNA Kit (Agilent). Sequencing libraries were prepared using Nextera DNA Flex Library Prep Kit (Illumina). Whole genome shotgun sequencing was performed on an Illumina MiSeq instrument using MiSeq Reagent Kit v.3 to generate 2 × 300 bp paired-end reads with an average of 2.8–3.1 million paired-end Illumina reads per genome. On average, the final coverage of the assembled genomes exceeded 200 × of the Illumina reads (Supplementary Table 1).

### Nanopore Data Generation

High molecular weight DNA was prepared using a modified phenol/chloroform protocol as previously described (Chiang-Ni et al., 2012). The gDNA was sheared using the Covaris g-TUBE (Covaris) to select fragment sizes ranging from 6 to 20 kb. The sheared gDNA was further selected using BluePippin with a 0.75% agarose gel cassette (Sage Science) to select gDNA fragment sizes ranging from 6 to 20 kb. Nanopore sequencing libraries were prepared using the PCR free, ligation-based sequencing kit (SQK-LSK109) with the native barcoding expansion (EXP-NBD104) for sample multiplexing. Nanopore sequencing was performed on an Oxford Nanopore MinION device (R9.4 flow cell FLO-MIN106D). In total, we obtained 161K–183K Nanopore reads for each strain and the average Nanopore read length was 8,875 bp and the L50 was 48,138 bp long.

### Genome Assembly and Annotation

The quality of the reads was evaluated using FastQC (v.0.11.9) (Andrews, 2010) and the low quality reads were subsequently removed by Trimmomatic (v.0.36) (Bolger et al., 2014) for the Illumina reads, and Nanofilt (v.2.6.0) (De Coster et al., 2018) for the Nanopore reads. The base quality of the Nanopore reads of greater than 1k bp was further improved by the corresponding high quality Illumina reads using FMLRC (v.1.0.0) (Wang et al., 2018). Taking advantage of the strengths of different algorithms in dealing with the repetitive regions, each strain was assembled by Canu (v.1.8) (Nurk et al., 2020), Flye (v.2.5) (Kolmogorov et al., 2019) and wtdbg2 (v.2.5) (Ruan and Li, 2020) individually to produce multiple versions of the draft genomes. These draft assemblies of each strain were then compared and merged by BLASTN (v.2.10.1) (Altschul et al., 1990) with manual inspection to produce a consensus assembly. The per-base accuracy was improved by Pilon (Walker et al., 2014) using the trimmed Illumina reads. On average, the final coverage of the assembled genomes exceeded 200 ×. The exact sequences of the duplicated TALE repeats and the large inverted repeats of plasmids were based on a second round of local *de novo* assembly using soft-clipped Illumina and Nanopore reads surrounding the draft *pthA* genes or the copper resistance gene cluster. The duplicated blocks were identified based on the proviso that the Illumina read coverage was more than two times higher than the background coverage of the corresponding plasmid sequence. The exact duplicated junction sites were then determined by high quality Nanopore long reads. Ambiguous regions where the sequencing depth was lower or higher than two times the standard deviation of the mean coverage were identified (Wang et al., 2021) and were locally reassembled and/or experimentally verified.

The genome sequences of the three strains were individually resolved into a single circular chromosome. All plasmid sequences were completely circularized as well (Supplementary Table 1). The genome annotation was performed by a local NCBI prokaryotic genome annotation pipeline (PGAP) (Lomsadze et al., 2018) with manual curation. In brief, the PGAP pipeline of the Docker image (v.2019-08-22.build3958) was used to perform the initial genome annotation. The assigned annotations were manually checked [Artemis (Carver et al., 2012)], e.g., correct start and stop codon), by comparative analysis with the published *X. citri* genomes (BLASTP (Altschul et al., 1990)).

### Whole Genome Comparison

Two complementary approaches based on the diversity of nucleotide sequences and protein coding genes were used for the genome-wide analysis to understand the overall genome variations. Assembled chromosome and plasmid sequences were compared with published *X. citri* genomes (Supplementary Table 2) to identify conserved and novel sequence elements. Whole genome sequence comparison was conducted by a series of bioinformatics tools including BLAST (v.2.10.1) (Altschul et al., 1990), fastANI (v.1.20) (Jain et al., 2018), Harvest (Treangen et al., 2014) and MUMmer (v.4.0.0rc1) (Kurtz et al., 2004). The whole genome similarity metrics of the three strains were estimated using the alignment-free approximate sequence mapping algorithm of fast Average Nucleotide Identity (fastANI) (Jain et al., 2018) with default settings. To further understand the origin of these three strains, whole genome sequences of 33 completely assembled *X. citri* strains (Supplementary Table 2) were included in the phylogeny analysis. The Parsnp algorithm in the Harvest suite (Treangen et al., 2014) used the maximum unique matches (MUMs) from the suffix graph data structure of the whole genome sequences to identify regions of the core-genome. The algorithm then performed multiple sequence alignments of multiple MUMs for subsequent variant calling and produced a SNP tree.

### Synteny Analysis, Pan-Genome, and Core Genes

The orthologous relationships between B2, T4 and SN3-3 was investigated. The protein coding genes of these three strains were searched using all-against-all BLASTP (Altschul et al., 1990). The BLASTP result was analyzed by a scalable unsupervised cluster algorithm TribeMCL (Enright et al., 2002) and the orthologous and paralogous relationships were presented as networks based on the *e*-values. The genome collinearity analysis was based on the genome synteny information calculated by i-ADHoRe 3.0 (Proost et al., 2012).

To further expand the analysis of gene repositories of *X. citri*, we compared protein coding genes of 79 *Xanthomonas* genomes (two *X. albilineans*, two *X. oryza* and 75 *X. citri*) (Supplementary Table 2) by BLASTP (Altschul et al., 1990) and TribeMCL (Enright et al., 2002) clustering. The homolog of the gene matrix in each genome was used as an input to calculate the average number of genes added with each additional genome using a method modified from Meric et al. (2014). Genes that were presented in more than one genome were considered as pan-genome and genes that were shared in at least two genomes were considered as core-genome. To obtain sufficient resolution of the phylogenetic positions, a total of 1,512 orthologous genes with a strict one-to-one relationship in 75 *X. citri* strains were used to construct the phylogenetic tree. In brief, protein sequences of single copy core genes were individually aligned by MUSCLE (Edgar, 2004) and gaps in the multiple sequence alignments were removed by Trimal (Capella-Gutierrez et al., 2009). Individual sequence alignments were concatenated to create a 478,302 amino acid-long sequence of each strain. Maximum likelihood phylogenetic trees were inferred by RAXML-NG (Kozlov et al., 2019) with 1000 bootstrap replicates (v.1.0.1, -model LG+G8+F –seed 2 –bs-trees 1000) and visualized by Figtree^1^.

### TALE Identification and CRISPR Analysis

The location and classification of TALEs were manually searched by BLASTN (Altschul et al., 1990) based on collected TALE sequences including short inverted repeats (IR), mobile insertion cassettes (MICs) and the Tn3 transposon *TnXax1* (Ferreira et al., 2015). Two short palindromic sequences and passenger genes were manually inspected as well. The structure of CRISPR and the Cas proteins were detected by CRISPRCasFinder (Couvin et al., 2018). CRISPRCasFinder integrated multiple tools to determine the hidden Markov model (HMM) profile of Cas proteins, the maximal repeat structure, the entropy of repeats, the sequence similarity and the size of spacers, and the sequence similarity with known CRISPR, and eventually assigned different levels of evidence code. The spacer sequences were further identified by BLASTN following the spoligotypes classification in Jeong et al. (2019). Phages and their integration sites were identified using PHASTER (Arndt et al., 2016) where published redundant phage/prophage sequences had been curated and the completeness scores were assigned to the identified phage regions.

### Copper and Arsenate Gene Cluster Analysis

The copper resistance gene clusters in the assembled plasmids were identified by comparison with known copper resistance genes in the plasmid pCuR (Gochez et al., 2018) and pLH201.1 (Richard et al., 2017a). Initial BLASTN hits were manually inspected and only regions with >60% sequence identity and >70% coverage of the query sequence were considered as the candidate area. Protein coding genes were then predicted using the PGAP pipeline (Lomsadze et al., 2018) and manually curated using Artemis (Carver et al., 2012). Comparative analysis results of gene clusters in pT4p2, pCuR and pLH201.1 were visualized by Artemis and Circos (Krzywinski et al., 2009).

### Horizontal Transfer of Copper Resistance Genes

Horizontal transfer of *cop* genes between different *X. citri* subsp. *citri* strains was tested according to the method in Behlau et al. (2012a). The strain T4 with copper resistance and rifampicin sensitivity was used as a donor. Spontaneous rifampicin resistant mutants of Cu^S^ strains B2Rif and SN3-3Rif were used as recipients. Bacterial strains were mated on NA plates at 28°C for 24 h. After mating, bacterial cells were scraped, suspended, and plated at dilutions on NA amended with rifampicin (50 mg/L) to estimate the population of the recipient. To select transconjugants, bacterial suspensions were plated at dilutions on NA supplemented with rifampicin (50 mg/L) and 0.8 mM CuSO_4_. The conjugation frequency was calculated as the ratio between the number of transconjugants and the population of the recipient (Behlau et al., 2012a).

## Results

### Complete Genome Sequence of Three *X. citri* Strains

The three strains of *X. citri* subsp. *citri* were able to cause similar levels of canker symptoms on Murcott leaves. No difference among the three strains was observed with regard to induction of symptoms. Furthermore, the strain T4 was characterized as a Cu^R^ strain which was able to grow on NA plates supplemented with 0.8 mM CuSO_4_ (Lai et al., 2021). The other two strains B2 and SN3-3, which could not grow on NA plates supplemented with more than 0.4 mM CuSO_4_, both showed sensitivity to copper.

To advance understanding of the genome structure and the molecular makeup of the Taiwan *X. citri* subsp. *citri*, we applied hybrid assembly by combining short read (Illumina) and long read (Nanopore MinION) sequencing technologies in an integrated bioinformatics workflow. Three type A strains, including two strains from one conventional commercial orchard (B2, copper sensitive and T4, copper resistant), and one strain from an orchard with minimal management (SN3-3, copper sensitive) were assembled into gap-free chromosome sequences (Supplementary Table 1).

**FIGURE 1:**
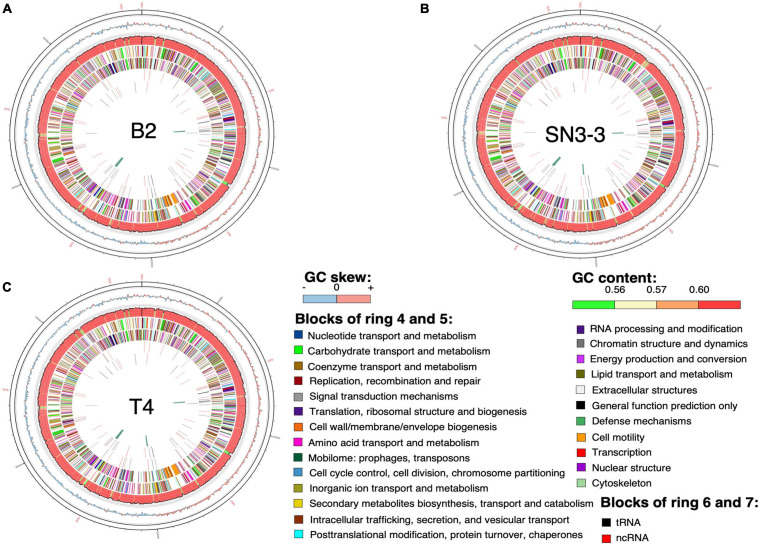
Chromosome organization and genome annotation. **(A–C)** Overview of three assembled chromosomes and the distribution of genetic elements. Circles from outermost to innermost represent: (1) position, the grid is 100 kb; (2) GC skew with 2500-bp sliding widow and 2500-bp step; (3) GC content in the same sliding window and step size as GC skew; predicted protein-coding sequence (CDS) with known function (4) of the plus strand and (5) the minus strand; predicted RNA of (6) the plus strand and (7) the minus strand; (8) phage sequence.

The chromosome sequences of the three strains were resolved into a single circular chromosome with length 5,120,747 bp (B2, accession number CP059999), 5,194,482 bp (T4, CP059992) and 5,192,310 bp (SN3-3, CP060002), respectively. To facilitate the comparative analysis, *DnaA* was organized in the beginning of the completely assembled genomes. The three conserved DnaA boxes of the *oriC* region could be identified between *DnaA* and *DnaN* (da Silva et al., 2002; Yen et al., 2002; Qian et al., 2005). The terminator of replication of each strain was located at around 2.5 Mb of the chromosome sequence. The replichore of the three strains could be identified based on the GC skew plot (Grigoriev, 1998). The assembled chromosome sequence length and GC content (64.76∼64.84%) (Figure 1) were in the same range as the published *X. citri* subsp. *citri* genomes (Timilsina et al., 2020). Through the genome assemblies, we also identified two plasmids in B2 (pB2_V1, 137,420 bp, CP060000; pB2_V3, 94,653 bp, CP060001), two plasmids in T4 (pT4p1, 99,130 bp, CP059993; pT4p2, 312,426 bp, CP059994) and one plasmid in SN3-3 (pSN3-3, 63,921 bp, CP060003). All plasmid sequences were completely circularized as well (Supplementary Table 1).

Using the NCBI prokaryotic genome annotation pipeline (PGAP) (Lomsadze et al., 2018) with manual curation, we identified 4,489 (B2), 4,557 (T4) and 4,539 (SN3-3) protein coding genes in the chromosomes with average coding capacity of 87.2, 87.1, and 87.1%, respectively. The leading strand and the lagging strand harbored a similar number of coding genes. Despite the *X. citri* reference genome 306 being published in 2002 and many genome sequences having been completed afterwards, gene function of a large part of the genome remains unknown. By comparison with the UniProt protein database and the manually collected *Xanthomonas* protein sequences from NCBI, ∼20% of the protein coding genes were predicted to be hypothetical genes. In addition to protein coding genes, all three strains contain 106 non-coding genes including 6 rRNA genes, 54 tRNA genes and 46 ncRNA genes (Supplementary Table 1).

### Low Nucleotide Sequence Diversity of Chromosomes

To further understand the similarity and possible origin of these three strains, we applied multiple complementary approaches to analyze the genome sequences. We first compared the chromosome sequence of the three strains to investigate the possible large scale chromosomal changes. The three strains shared high sequence identity and collinearity (Supplementary Figure 1). We then examined the nucleotide sequence divergence at the whole genome level. Instead of using the conventional approach for calculating average nucleotide identity (ANI) of orthologous genes, we applied an alignment-free, whole-genome average nucleotide identity (FastANI) analysis (Jain et al., 2018) to determine the pairwise ANI values. The three strains sequenced in this study were found to share very high levels of sequence identity (>99.96%) and there were no significant differences among them (Supplementary Table 3). By focusing on the orthologous chromosome sequences conserved in these three strains and 33 assembled *X. citri* strains in the same pathovars (Supplementary Table 2), we used Parsnp in the Harvest package to perform the core-genome alignment (Treangen et al., 2014). *X. citri* strains of the same pathotype were grouped in the same phylogenetic clade (Supplementary Figure 2). Three strains sequenced in this study, B2, T4 and SN3-3, were all classified into the pathotype A. However, these strains were respectively grouped with strains from different geographic origins (Supplementary Figure 2). In particular, T4 shared higher sequence similarity with the reference strain A306 from Brazil and the other strains from Argentina whereas B2 was closer to the strains from Jiangxi, China. SN3-3 was closer to strains from Jiangxi and Guangdong, China.

### Spoligotype Classification by CRISPR Spacer Array

**FIGURE 2:**
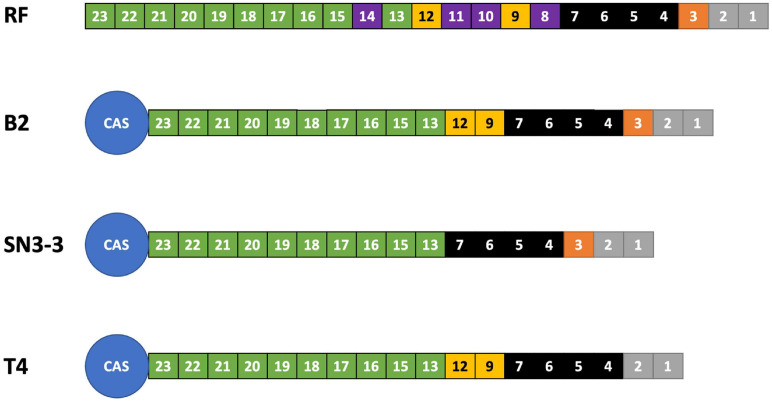
CRISPR spoligotyping. Overview of the CRISPR/Cas locus. The reference (RF) array annotation is based on *X. citri* 306 (CP009016). The colors of the boxes indicate spacer sequences that correspond to the reference. Purple: absent in the sequenced genomes of this study.

We analyzed the compositions and orders of CRISPR and spacer array of our strains by CRISPRCasFinder (Couvin et al., 2018). Following the presence and absence of spacer patterns (spoligotypes, spacer oligonucleotide) (Jeong et al., 2019), B2 contained 19 spacer/repeat units, T4 contained 18 units and SN3-3 contained 17 units, corresponding to spacer Xcc_01 to Xcc_23; spacer Xcc_8, Xcc_10, Xcc_11 and Xcc_14 were absent in these strains (Jeong et al., 2019). In particular, B2 and SN3-3 shared a common Xcc_3 whereas Xcc_9 and Xcc_12 were only seen in B2 and T4 (Figure 2). According to the spoligotype classification by Jeong et al. (2019), B2 belongs to spoligotype 8 (China, Florida, Reunion islands), T4 belongs to spoligotype 14 (Brazil, Mali) and SN3-3 belongs to the spoligotype 21 (Japan). The CRISPR spacer arrays demonstrated that our strains of the same pathotype A belonged to the different spoligotypes originally from East Asia, the Indian Ocean, and South America.

### Core Genome Phylogeny

Orthologous genes were first identified by an all-against-all BLASTP search and the BLASTP result was subsequently analyzed by the Markov chain based TribeMCL (Enright et al., 2002) to divide them into orthologous groups. The orthologous information was then used by the synteny based gene family analysis (Proost et al., 2012) to determine genes in the collinearity region. In total, 4,090 orthologous genes were shared between the three strains and did not show chromosome rearrangements (Supplementary Figure 1). Around 144–237 genes are strain specific and were found in only one of the genomes (Supplementary Figure 3). Detailed functional analysis by GO enrichment of these strain specific genes indicated that most of them were enriched with transposon activity-related genes (Supplementary Table 4). This result indicated that the chromosome variations between the three strains, though minimal, were likely associated with the transposon activities.

**FIGURE 3:**
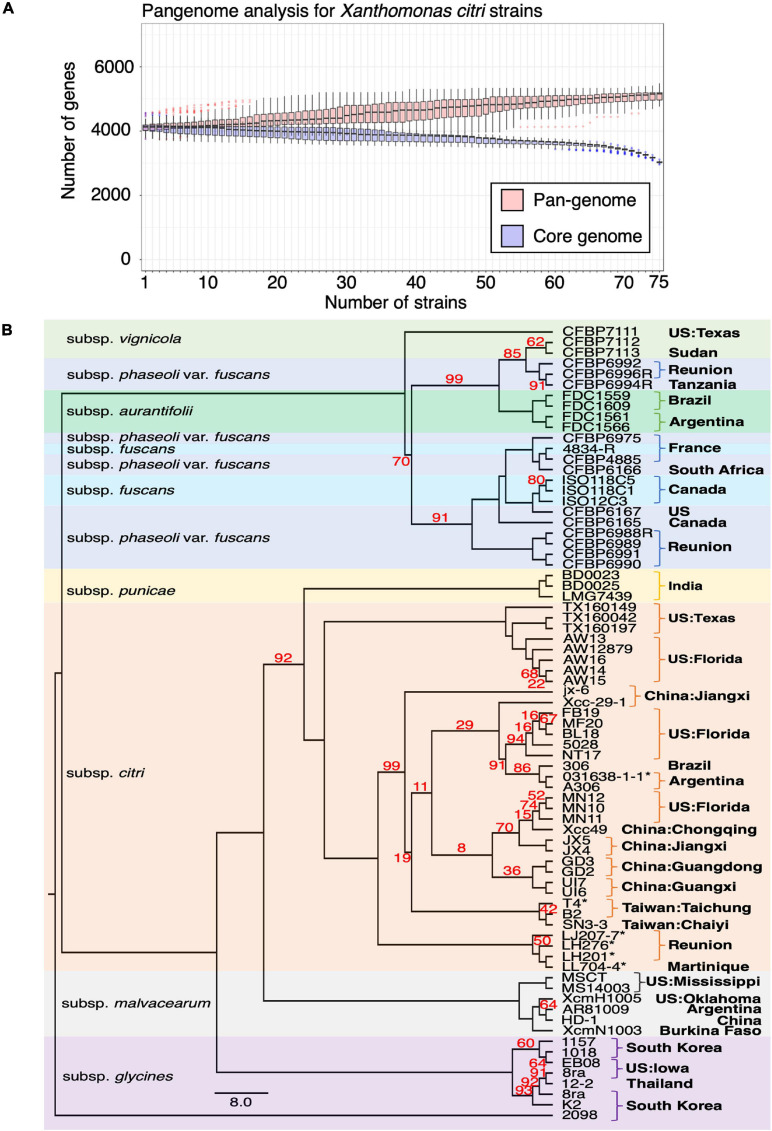
Pangenome analysis of Xanthomonas genomes. **(A)** Pangenome analysis of 75 *X. citri* genomes. **(B)** The core genomes of 75 *X. citri* sp. strains showed a phylogenetic relationship and shared geographic origins. Maximum likelihood phylogeny based on concatenated sequences of 1,512 core genes. The asterisks (*) indicate copper-resistant strains, and branches with bootstrap value < 100 support are labeled with red numbers.

To better understand the chromosome dynamics of *X. citri* strains, we conducted pan-genome analysis with protein coding genes of our three sequenced strains in this study and 76 published *Xanthomonas* genomes. Orthologous genes of 79 strains (including two *X. albilineans*, two *X. oryza* and 75 *X. citri*) (Supplementary Table 2) were determined by the combination of an all-against-all BLASTP search and TribeMCL clustering. We identified 6,342 ortholog clusters that were shared between more than two strains. That is, 99.7% of the genes in one strain were shared in at least one of the 78 sequenced strains. Within the 79 *Xanthomonas* genomes used for the comparison, the pan-genome size was around 5,000 genes and the core-genome size was less than 2,000 genes (Timilsina et al., 2019; Supplementary Figure 4). Nevertheless, when four outgroup species (2 *X. albilineans*, 2 *X. oryza*) were removed from the analysis, the pan-genome size remained around 5,000 genes but the core-genome size increased to ∼3,000 genes (Figure 3A). The number of core genes identified in our study is comparable with those obtained from 58 *X. perforans* strains (Timilsina et al., 2019).

We used the phylogenomics approach to further understand the phylogenetic positions of three strains in this study and other published genomes. Based on the core genome analysis, we selected 1,512 orthologous genes that had a strict one-to-one single copy relationship to construct the phylogenetic tree. A tree inference tool based on the maximum likelihood (ML) method (Kozlov et al., 2019) was used to construct the phylogenetic tree. As expected, the strains of different pathotypes were clustered on different clades of the phylogenetic tree with high bootstrap value support for each lineage (Figure 3B) and correspondence to the origin of the collection. The pathotype A strains including the reference strain 306 and our three strains were placed together in the same clade together with strains from China, Florida, Argentina and Brazil. On the other hand, four strains from Reunion were placed in an independent clade (Richard et al., 2021).

### Type II and Type III Secretion Systems

In *Xanthomonas*, the type II secretion system (T2SS) is used to translocate folded proteins from the periplasm into the extracellular milieu (Korotkov et al., 2012) and the type III secretion system (T3SS) is essential to pathogenicity through modulating host plant physiology and enabling evasion of host immune responses (Timilsina et al., 2020). In the T2SS, we identified two operons, each containing eleven genes of the general secretory pathway (Gsp) (Korotkov et al., 2012) as was previously reported in the reference strain 306 genome (da Silva et al., 2002; Supplementary Figure 5A and Supplementary Table 5). *GspO* could be identified in the chromosome but is not located in the two Gsp operons. Gene clusters encoding for *hrp* (hypersensitive response and pathogenicity), *hrc* (*hrp* conserved) and *hpa* (*hrp* associated) of the T3SS (Alegria et al., 2004) were identified in the chromosome (Supplementary Figure 5B and Supplementary Table 5). Overall, the T2SS and T3SS were highly conserved in the three sequenced strains.

### TALE Variation and Plasmid Fusion

Compared with the chromosome sequences, the plasmid sequences showed higher diversity in plasmid length and number of genes (Supplementary Figure 6; Timilsina et al., 2020). In total, we identified five plasmids in three strains. The plasmid size ranged from 64 to 312 kb and the GC content was around 60%, which was lower than the average of 64% of the chromosomes (Supplementary Table 1). We tried to determine the plasmid copy number in each genome based on the relative whole genome sequencing coverage with the chromosome (Pena-Gonzalez et al., 2018). On average, we estimated the genomes contained 2.6, 3.6, 4.4, 3.5, and 1.6 copies of pB2_V1, pB2_V3, pSN3-3, pT4p1, and pT4p2, respectively (Supplementary Figure 7).

**FIGURE 4:**
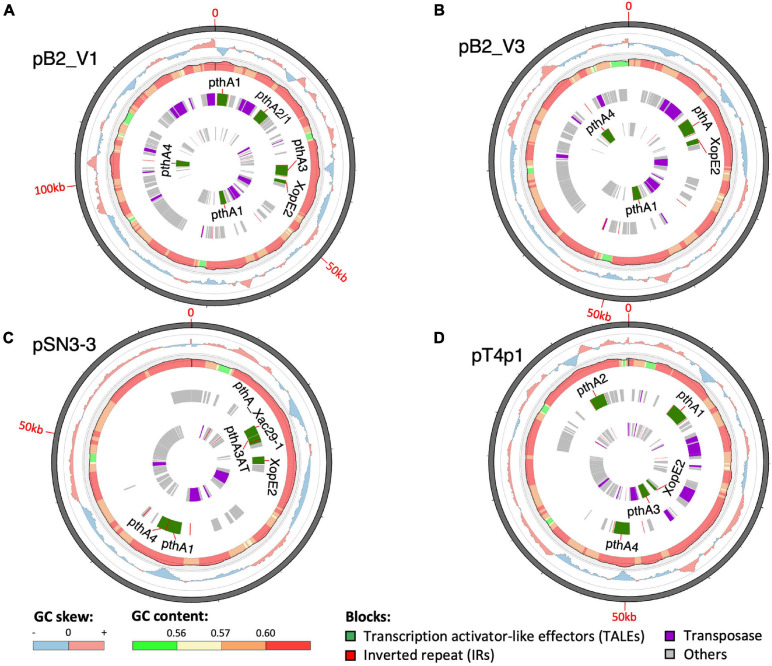
Plasmid organization. **(A)** pB2_V1, **(B)** pB2_V3, **(C)** pSN3-3, and **(D)** pT4p1. Circles from outermost to innermost represent: (1) GC skew, (2) GC content, (3) reverse, and (4) forward direction of repetitive sequences. Green: TALEs. Red: inverted repeats. Purple: transposase. Gray: other CDS/genes: gray.

A sequence similarity search of plasmid sequences by BLASTN indicated pT4p2 contained the copper resistance gene cluster as indicated in Gochez et al. (2018). The other four plasmids (pB2_V1, pB2_V3, pSN3-3, and pT4p1) sharing high similarity with known plasmid sequences showed a high level of sequence rearrangement (Figure 4 and Supplementary Figures 6, 8). For instance, mobile genetic elements including Tn3-like transposon and the subclass of insertion sequence (IS) elements were highly variable between the four pathogenicity-related plasmids. Analysis of repeat-variable diresidues (RVDs; Ferreira et al., 2015) indicated that TALE repeats were seen in these four plasmids which contained different copy numbers of *pthA* encoding genes with various sizes of TALEs (Supplementary Table 6). Three copies of *pthA* gene were found in strains SN3-3, four copies in T4 and eight copies in strain B2.

**FIGURE 5:**
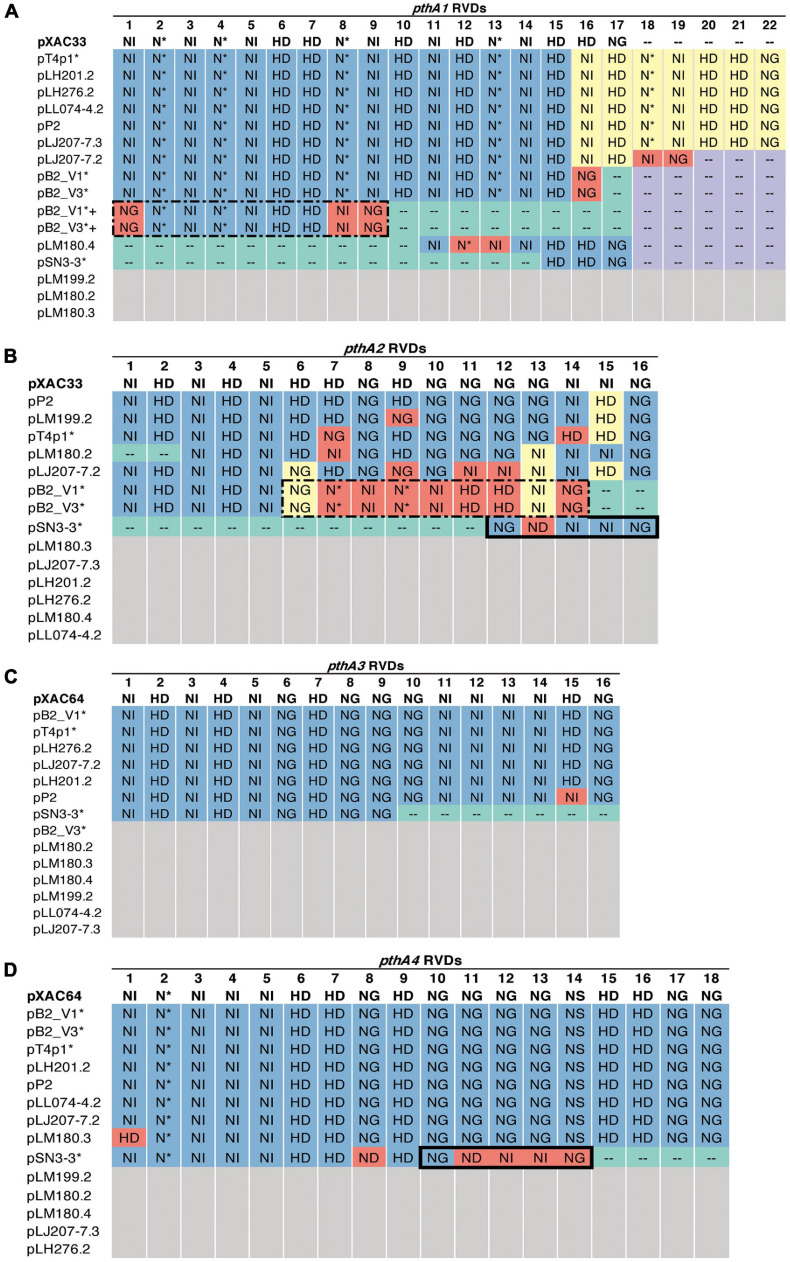
RVD profile of *pthA* genes. The asterisks (*) indicate plasmids sequenced in this study. NI (Asn-Ile) recognizes A; HD (His-Asp) recognizes C (but not 5′-methyl-C); NG (Asn-Gly) recognizes T and 5′-methyl-C; NN (Asn-Asn) recognizes G or A; NS (Asn-Ser) recognizes A,T,C or G; N* (Asn-*) recognizes C or T [47]. Blue: identical to the reference. Purple: absent, the same as the reference. Green: absent, different from the reference. Yellow: > 50% sequence similarity with the reference. Red: unique. White: no such *pthA* genes. **(A)** Plus sign (+): merged *pthA* genes. **(A,B)** Dashed box: nine *pthA1* repeats were merged with *pthA2* repeats. **(B,D)** Solid box: five *pthA2* repeats were merged with *pthA4* repeats.

Based on the TALE classification, Class I contained *pthA2* and *pthA3* genes, Class II contained *pthA4* and Class III contained *pthA1* (Gochez et al., 2018). These three Classes were seen together in plasmids pSN3-3, pB2_V1, and pT4p1 (Figure 4) and represented the signature of plasmid fusion events (Figures 5, Supplementary Figures 6, 8, and Supplementary Table 6). The size of pSN3-3 is similar to that of pXAC64 of the reference strain 306 but the *pthA* genes of strain SN3-3 carry different copies of repeats (Supplementary Table 6), which are shorter and not comparable with those of the reference strain 306 (da Silva et al., 2002). On the other hand, *pthA2* and *pthA4* on pSN3-3 partially overlapped with each other (Figure 5 and Supplementary Table 6).

Plasmid pT4p1 was highly similar to plasmid pP2 of the Cu^R^ strain Xc-03-1638-1-1 (Supplementary Figure 6 and Supplementary Table 6; Gochez et al., 2018). The two plasmids were almost identical with only 23 bp difference and contained the exact copy number of all four classes of *pthA1* genes. Plasmid pT4p1 had 4 *pthA* genes of 3 classes including two genes with 15.5 (Class I: *pthA2* and *pthA3*), one with 17.5 (Class II: *pthA4*) and one with 21.5 (Class III: *pthA1*) repeats comparable with the second largest plasmids of Cu^R^ strains pLH276.2 and pLL074-4.2 (Gochez et al., 2018; Supplementary Table 6). The plasmids of strain B2 had 8 *pthA* genes of 3 classes including two genes with 13.5 (unclassified class: *pthA2/1*), three with 15.5 (Class III) and two with 17.5 (Class II) repeats partially comparable to the pathogenicity plasmids of Cu^R^ strains (Gochez et al., 2018; Supplementary Table 6). The effector genes *xopE2* (Ferreira et al., 2015) and *pthA4* (Roeschlin et al., 2019) that are involved in the suppression of the hypersensitive response of the host were also identified in these four pathogenicity-associated plasmids.

### Structures and Origins of the Copper and Arsenate Resistance Gene Clusters

**FIGURE 6:**
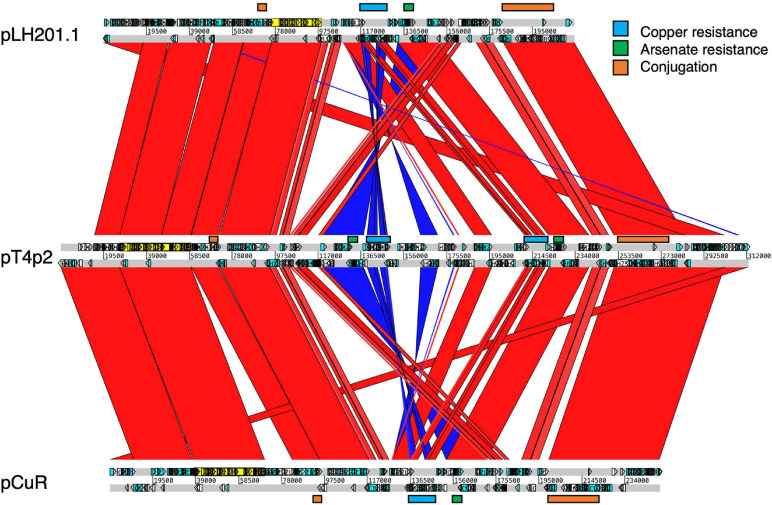
Comparison of plasmids containing copper resistance genes in pT4p2, pCur and pLH201.1. Syntenic regions are presented in red color and inverted regions are in blue. Genes involved in copper/arsenate resistance and conjugation are highlighted along the plasmid sequences.

**FIGURE 7:**
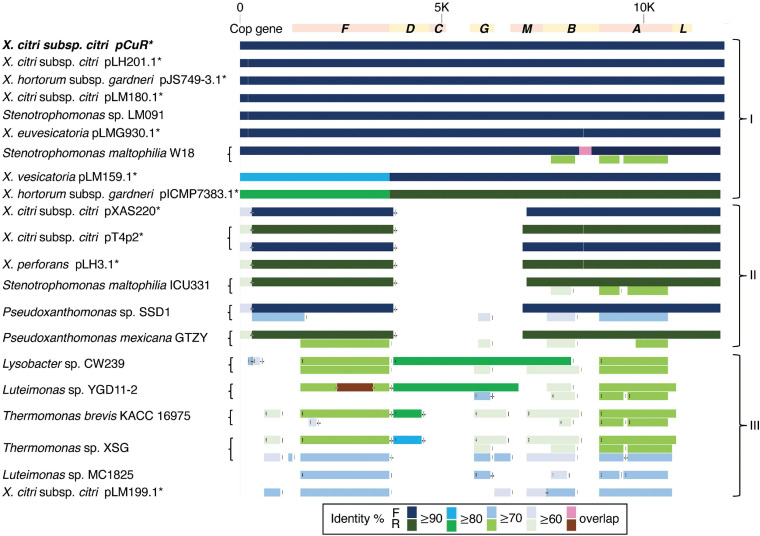
Comparison of gene gain and loss in the copper resistance gene cluster.

The Cu^R^ strain T4 had the largest plasmid pT4p2 among the three sequenced strains (Supplementary Table 1) for which the sequence is similar to the copper resistance plasmids pLH201.1 and pCuR (Figure 6). Interestingly, the sequence of pT4p2 is larger than those two plasmids due to an unusual ∼40 Kbp inverted repeat. We further confirmed the junction size of the inverted repeat by PCR (Supplementary Figure 9). On the other hand, pT4p2 shared the same inverted repeat pattern as observed in pLH3.1 of *X. perforans* LH3 (CP018472) (Supplementary Figure 10), where the strain LH3 was collected from a tomato orchard in Mauritius in 2010 (Richard et al., 2017b). Each inverted repeat contains a complete set of the copper resistance gene cluster, except *copCDG* are missing (Figures 6, 7). Analysis of *cop* gene clusters revealed that three groups of copper resistance gene clusters could be identified across a diverse set of bacterial species. pLH201.1 and pCuR belonged to Group I. The pT4p2 and copper resistance plasmids from *X. perforans*, *Stenotrophomonas*, and *Pseudoxanthomonas* sharing the same arrangement of the *cop* gene cluster belonged to Group II (Figure 7 and Supplementary Table 7). In particular, in the NCBI nucleotide database, a unique region between the copper-arsenate clusters was only identified in pT4p2, pLH3.1 and the plasmids of six other *Xanthomonas* strains that were tomato pathogens, with the exception of pXAC219 that was isolated from the citrus leaf. Plasmids of these strains shared 99% sequence coverage and >96% sequence identity (Supplementary Table 8). The third group, Group III, contained a more diverse set of *cop* gene clusters where the plasmid backbone was extremely similar to pCuR (Richard et al., 2017b) but only *copF* and *copA* shared 70% nucleotide sequence identity with those in pCuR.

**FIGURE 8:**
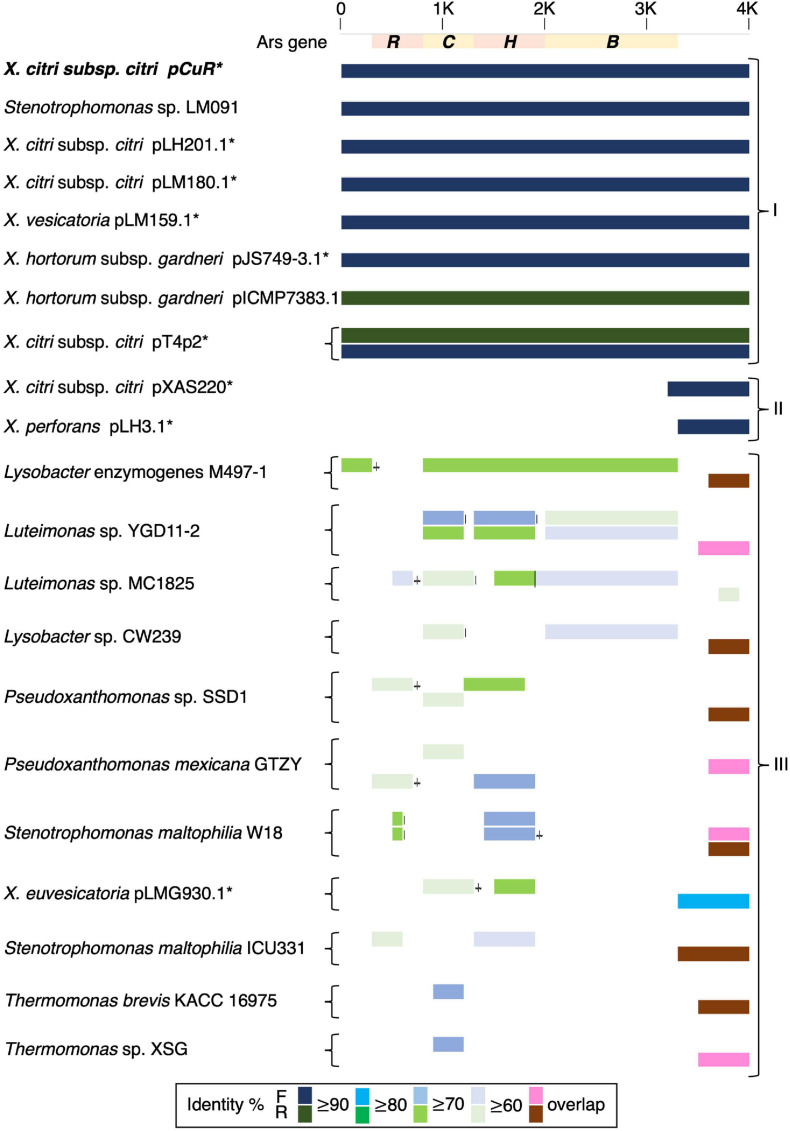
Comparison of gene gain and loss in the arsenate resistance gene cluster.

To further support our idea about the origin of the copper resistance cluster, we then compared the structure of the arsenate gene cluster. The arsenate cluster was mostly composed of either three (*arsRBC*) or five (*arsRDABC*) genes and expressed in a single transcriptional unit in soil bacteria (Achour et al., 2007). Interestingly, the *X. citri* arsenate gene cluster which was grouped together with that of *Stenotrophomonas* was composed of four (*arsRCHB*) genes (Figure 8). Furthermore, the transposases between the arsenate and copper clusters showed higher sequence similarity to those of *X. perforans* whereas two endonucleases corresponding to pCuR and pLH3.1 were adjacent to each other (Supplementary Figure 10).

Moreover, the *ars* and *cop* gene clusters responsible for resistance to heavy metals including copper and arsenate are adjacent to each other with higher GC content (62.3%) compared with other genomic regions (58.6%) and flanked by two Tn3-like transposons (Figure 6 and Supplementary Figure 11). In addition to the abnormal GC content and transposon insertions, *merR* and *cusAB* associated with heavy metal resistance in bacteria were found in the pT4p2. The MerR transcriptional regulator family controlled the expression of *copA* in *Escherichia coli* and responded to environmental stimuli, such as heavy metals or antibiotics (Brown et al., 2003). In pT4p2, *MerR* is 7 Kbp away from the *ars* cluster but the heavy metal efflux pump *cusAB* is next to the *ars* cluster (300 bp). The close proximity of *MerR* and *cusAB* to the heavy metal cluster was also observed in pLH201.1 (Richard et al., 2017b). In addition, the heavy metal resistance gene clusters in pT4p2 were surrounded by two *Tra* gene clusters. The T4 strain showed the conjugation ability and the pT4p2 encoded 16 Tra proteins that are essential for conjugation. Similar to the pCuR and pLH201.1 arrangement, *Tra* genes of pT4p2 located in two different regions of the plasmid with *TraID* in one side and other 14 *Tra* genes grouped into another cluster. Overall, the complete set of *Tra* genes in the pT4p2 and the high sequence similarity of heavy metal resistance gene clusters with other published plasmids indicated that the heavy metal resistance gene was likely acquired from other microbes.

### Conjugative Transfer of Copper Resistance Genes

The copper resistance genes can be transferred from the Cu^R^ strain T4 to the Cu^S^ strains B2Rif and SN3-3Rif through bacterial conjugation (Supplementary Table 9). The transconjugants showed the same level of copper resistance as the donor strain T4. The frequency of conjugative gene transfer ranged from 10^–8^ to 10^–5^ transconjugants per recipient (Supplementary Table 9). B2Rif received the Cu^R^ genes from T4 more frequently than SN3-3Rif (Supplementary Table 9).

## Discussion

In this study, the genome and plasmid sequences of three *X. citri* subsp. *citri* strains from Taiwan were completely sequenced and assembled using the combination of Illumina short-reads and Oxford Nanopore long-reads. Whole genome sequencing has been widely used to understand the pathogenic, taxonomic and phylogenetic status of the xanthomonads (Zhang et al., 2015; Bansal et al., 2017; Patane et al., 2019). Previous studies lacked the resolution of repetitive or duplicate regions as they could not be precisely resolved by the short-read sequencing method and the bioinformatic algorithm (Triplett et al., 2011; Fonseca et al., 2019). Recently, with the availability of the long-read sequencing method and the advances in genome assembly (Kolmogorov et al., 2019), complex genome structures can be fully resolved. Correspondingly, the genome and plasmid sequence dynamics of *X. citri*. from different geographic locations have gradually emerged (Gochez et al., 2018; Roach et al., 2020; Richard et al., 2021).

The assembled chromosome sequence length, GC content (64.76∼64.84%) and number of genes (Figures 1A–C) are in the same range as the published complete *X. citri* subsp. *citri* (Timilsina et al., 2020). The 75 *X. citri* genomes showed a high level of genome conservation where 2,529 ortholog genes had a strict one-to-one single copy relationship within *X. citri* strains. The large core-genome size (Figure 3A) and the pangenome analysis (Loiseau et al., 2018) indicated that the *X. citri* strains showed a ‘close’ genome signature where strains shared a large common gene repertoire. In contrast, the *Xanthomonas* genus was an ‘open’ genome where gene gain and loss events frequently occurred within different *Xanthomonas* lineages (Timilsina et al., 2020).

Comparing chromosome sequences and gene contents did not reveal many differences between *X. citri* subsp. *citri* strains. In fact, the pangenome analysis confirmed that strains in the *X. citri* subsp. *citri* were rather conserved and the core genome was composed of 2,529 one-to-one ortholog genes and ∼3,000 genes (i.e., detected in all 75 strains) (Figure 3A). Nevertheless, the core genome size of this study is smaller than the 4,347 genes of 221 strains in the previous study (Richard et al., 2021). The differences in the three pan-genome analysis strategies likely explain the inconsistency between the studies. First, only protein coding genes in the chromosomes were considered for the orthologous relationships in this study (Timilsina et al., 2019); however, the study by Richard et al. included the plasmid genes in the analysis (Richard et al., 2021). A high gene turnover rate (gain or loss of genes) was observed due to uneven frequencies of acquiring or losing a single large plasmid in different lineages (Richard et al., 2021). The ease of horizontal gene transfer of the entire plasmid between strains has made plasmids the main driver of host adaptation (Ruh et al., 2017). To focus on changes in the chromosome sequences, we decided to focus only on genes of the chromosomes.

Secondly, we used orthology-based analysis where predicted protein coding genes of each of the *de novo* assembled genomes were used to identify orthologous genes among sequenced strains. The advantage of this strategy is that it did not restrict the analysis in one particular reference strain. That is, unique genome fragments and gene contents that existed in one particular strain could be identified. The main drawback was that each sequencing project applied different gene prediction methods and this might introduce biases in gene models due to technical artifacts. An alternative approach would be based on the read coverage of the whole genome resequencing data (Richard et al., 2021). Using this approach, one strain was selected as the reference genome where resequencing reads were mapped to the reference genome sequence. The presence and absence of the gene was then evaluated based on the pre-defined cut-off of read depth and coverage of the gene length. This method could avoid the fragmented genomes and truncated gene models due to the short read assemblies. Nevertheless, variations due to paralogs, gene copy number or pseudogenization could not be identified. Despite the differences in the exact number of genes of the core genome, our result agrees with previous analysis (Timilsina et al., 2019) in concluding that *X. citri* subsp. *citri* is a monomorphic bacterium and the gene numbers are conservative in the lineage (Timilsina et al., 2019).

The pathotype classification is in agreement with the core-genome phylogenetic analysis in showing that these three strains clustered with the pathotype A strains from different geographic origins (Jeong et al., 2019). However, the exact phylogenetic relationship between the three strains sequenced in this study could not be clearly resolved and we did not date the time scale of the evolutionary history (Figure 3B). The three strains were placed in an independent clade with 100% bootstrap value support. The long branch length with strains from other geographic origins suggest that three strains diverged from a common ancestor.

The gap-free complete genome sequences offered a unique opportunity to study the structure of the repetitive sequences such as CRISPR repeats and spacers in detail since their order and the exact copy number have often been mis-assembled in the draft genome assemblies (Wang et al., 2021). In particular, spoligotyping provided an alternative method to infer the evolutionary history and the common ancestor of the microbial strains. Among sequenced *X. citri* subsp. *citri* strains, the CRISPR array contains a conserved array of 23 spacers and could be used to infer the evolutionary trajectory among the observed spoligotypes (Jeong et al., 2019). This method was based on the principle associated with the order of the CRISPR array, where the *cas* genes tend to be associated with more recently acquired CRISPR repeats. For instance, spacer Xcc_23 is likely to be a newly acquired spacer/repeat unit where spacer Xcc_1 might be the oldest spacer (Jeong et al., 2019). The results of the CRISPR array are consistent with the notion that *X. citri* originated from a common ancestor and formed a monophyletic clade (Bansal et al., 2018). Furthermore, strains B2 and T4 from the commercial orchard under conventional management have more CRISPR repeats than the strain SN3-3 which was from the orchard with minimal management. This result implied that the number of CRISPR repeats could be affected by types of agricultural management. Furthermore, B2 and T4 originated from the same orchard but differed in composition of CRISPR repeats. Xcc_3 was found in B2 but not in T4 (Figure 2). Thus, *X. citri* subsp. *citri* in an orchard could evolve into various strains with diverse compositions of CRISPR repeats. The spoligotyping data indicated the rapid evolution and diversity of *X. citri* subsp. *citri* pathotype A in Asia.

The function of CRISPR/Cas systems has been proven to be an adaptive bacterial defense system against phage infections. New CRISPR spacers are introduced in the bacterial chromosome near the leader sequence (Datsenko et al., 2012). Yet, frame-shift mutation of *csd1*/*cas8c* genes caused by a short tandem repeat of two base pairs (AG) was found in the majority of *X. citri* strains, suggesting that the CRISPR defense system was mutationally inactivated to further acquire new spacers (Jeong et al., 2019). The results also suggest that most of the 25 spoligotype patterns found in *X. citri* could evolve by either random deletion of a single spacer/repeat unit or simultaneous deletion of adjacent spacer/repeat units (Jeong et al., 2019). Accordingly, deletion events could occur more often in SN3-3 than in the other 2 strains since SN3-3 had fewer CRISPR repeats than B2 and T4. An extra AG is also present in the *csd1*/*cas8c* genes of our *X. citri* subsp. *citri* strains (Supplementary Figure 12) reflecting inactivation of the CRISPR system due to frame-shift mutation. Moreover, the CRISPR repeat profile data implied that B2 and T4 could acquire more resistance to phage infections than SN3-3. It will be intriguing to explore whether minimal agricultural management could induce more deletion of CRISPR repeats in citrus canker pathogens or reduce diversity of phages in an orchard.

Plasmids are highly dynamic and present another source of the *X. citri* genome diversity. The number and size of plasmids vary between *X. citri* strains and are important contributors to pathogenicity due to their ease of acquisition of foreign genomic elements through recombination and horizontal gene transfer (Timilsina et al., 2020; Rodriguez-Beltran et al., 2021). Mobile genetic elements are known to facilitate the exchange or transfer of genomic fragments between chromosomes and plasmids (Blair et al., 2015). Metagenomic analysis of wastewater identified that antibiotic selection pressure significantly increased the abundance of antibiotic resistance genes, reduced the diversity of the microbial community and in particular, increased the occurrence and abundance of mobile genetic elements (Zhao et al., 2021). Despite the high sequence variability, genes in several important functional categories such as conjugative transfer (*traY*, *traD* and *mobD/A/L*), the type IV secretion system (*vir2/3/4/6/8/9/11*), toxin/antitoxin system (family *vapC* or *pemK/mazF*) and core genes (*repA*, *parA*, XRE family transcriptional regulator) were found in all of our plasmids (Gruber et al., 2016).

Multiple types of insertion sequences and transposons including IS3, IS4, ISxac3, ISxac2, TnpA and Tn3 were identified in the plasmids (Figure 4). The xanthomonad Tn3 family transposons were located near different TALEs. The TnXax1 transposable element of Tn3 family in xanthomonadaceae were flanked by short inverted repeat (IR) sequences forming a generic structure of mobile insertion cassettes (MICs; Gochez et al., 2018). In particular, the genetic content of TnXax1 was organized in the following order from left IR (IRL) to right IR (IRR): *mlt* + *TnpA* (transposase) + *TnpR* (resolvase)/*TnpS*/*TnpT* (recombinase) + passenger gene as observed in other Xanthomonas strains (Lima-Mendez et al., 2020). The structure of the MICs in the plasmids of this study was similar to *TnXax1* containing the same passenger genes, Tn3 transposons and inverted repeats (IRs; Ferreira et al., 2015). The *pthA* genes in the four pathogenicity-related plasmids were surrounded by the same IRR and IRL as in the pXAC64 plasmid of *X. citri* 306.

TALEs are located on plasmids in *X. citri* strains with high variability in order to adapt to different hosts (Ferreira et al., 2015; Timilsina et al., 2020; Figures 4, 5 and Supplementary Table 6). The variability of the TALE repeats has been considered to be a strategy to diversify selection pressure to escape the detection of the host R genes (Doucoure et al., 2018). The changes in repeat arrays in *X. oryzea* TALEs were mainly associated with repeat deletion, and recombination with other TALEs (Teper and Wang, 2021). Furthermore, Teper and Wang (2021) demonstrated that two to five mismatched TALE repeats of *X. citri* subsp. *citri* was sufficient to escape the host NB-LRR recognition and promote disease symptoms in sweet orange. Our data revealed that most *pthA4* genes from different *X. citri* subsp. *citri* strains have the same number and RVD of tandem repeats (Figure 5 and Supplementary Table 6). Surprisingly, the *pthA4* of strain SN3-3 was not only short (13.5 repeats) but also merged with *pthA2*.

It has been estimated that the optimal functional length of TALEs contained 15.5–19.5 RVD repeats whereas TALEs with fewer than 6.5 repeats did not perform gene activation and could be by-products of recombination events (Boch and Bonas, 2010; Gochez et al., 2018). Nevertheless, some ‘non-classical’ TALEs with unusually short lengths of RVD repeats but that maintain their biological function, have been reported recently (Roeschlin et al., 2019). We also identified a fusion of *pthA1* and *pthA4* in pSN3-3 and *pthA1* that was surrounded by a solo IR sequence containing only 2.5 RVD repeats (Figure 5 and Supplementary Table 6). The *pthA4* was indispensable for canker elicitation but *pthA1* and *pthA3* could contribute to additive roles in developing disease symptoms (Abe and Benedetti, 2016). Since PthA4 is the TALE required for pathogenicity and SN3-3 was collected from a citrus farm with minimal management, we speculated that the PthA4 with 13.5 RVD repeats was sufficient to develop pathogenicity and maintain bacterial fitness under the agroecosystem with low selection pressure. Our data is in full agreement with the notion that the presence of the Tn3-like transposons around TALEs likely contributed to the generation of diverse TALEs of *X. citri* (Ferreira et al., 2015).

The genomic signatures of the three strains supported the occurrence of independent events of plasmid fusion. Compared with the reference genome of strain 306 (da Silva et al., 2002), we were able to identify the recombination event that caused the plasmid co-integration and the fusion of *pthA* genes (Figure 5). Unlike different *pthA* classes that were separated in two plasmids in pXAC33 and pXAC64 (da Silva et al., 2002), pB2_V1 and pT4p1 each contained three *pthA* classes in one plasmid (Supplementary Table 6). Furthermore, pB2_V1 contained two copies of *pthA1* class with high sequence identity and pB2_V1 and pB2_V3 both contained a unique class of *pthA2*/*pthA1* fusion with 13.5 RVD repeats (Figures 5A,B and Supplementary Table 6). The plasmid size of pSN3-3 was similar to pXAC64 of strain 306 (da Silva et al., 2002) but also had three classes of *pthA* and the fusion of *pthA1*/*pthA4* in one plasmid (Figures 5B,D).

The whole genome sequence of T4 clarified that the *cop* gene cluster is borne on the big plasmid pT4p2. Though the copper resistance in different *Xanthomonas* strains might be acquired through independent events, *cop* genes in the plasmids were genetically related (Richard et al., 2017b) and the backbones of the plasmids are highly similar. Based on the nucleotide sequence identity and presence and absence of *cop* genes, we could divide plasmids, based on the copper cluster, into three groups (Figure 7). Group I, including the reference plasmid pCuR and pLM091 from *Stenotrophomonas* (Richard et al., 2017b), contained the complete set of *copLABCDMGF* genes without gaps and shared high sequence identity (>90%) with plasmids in this group. On the other hand, the most dissimilar group (Group III), including pLM199 from Argentina, had sequence identity with pCuR that was ∼70% and failed to produce PCR amplicons using primers of the *copLAB* system (Richard et al., 2017b). A distinct copper transposon region (TnpLM199) containing an alternative copper resistance system *copABCD* was identified in the Argentinian strain LM199 genome (Richard et al., 2017b).

The pT4p2 was classified in Group II together with pLH3.1 of *X. perforans* and chromosome sequences from other microbials. In particular, a unique nucleotide region between the copper and arsenate cluster was only identified in pT4p2 and pLH3.1. A detailed analysis of the 1,857 bp spacer nucleotide sequence between the copper and arsenate cluster in the NCBI Nucleotide database revealed that only seven *Xanthomonas* strains contained this fragment. Among these seven strains, the spacer sequence was not located on the same plasmid as that containing copper resistance genes in the *X. euvesicatoria* strain LMG930. This result is in agreement with a previous analysis that revealed that the *copB* genes carried by pT4p2 and LMG930 were grouped in the ‘Variant IV’ group and shared a combination of 3, 300 and 36 bp gaps in the complete *copB* sequences (Lai et al., 2021). On the other hand, the other six *Xanthomonas* strains showed high levels of sequence coverage (99%) and nucleotide identity (>96%) (Supplementary Table 8). In particular, pLH3.1 of *X. euvesicatoria* did not only show high identity of the spacer sequence but also the *cop* genes (Supplementary Figure 10). Accordingly, the comparative genome analysis data provided evidence that pT4p2 could originate from pLH3.1 carried by Mauritian *X. perforans* LMG930. Although Cu^R^ *X. euvesicatoria* pv. *perforans* populations have prevalently occurred in domestic tomato orchards in Taiwan since 1989 (Burlakoti et al., 2018), only the variant V group of the *copB* gene was carried by *X. euvesicatoria* pv. *perforans* strains in our recent survey (Lai et al., 2021). Thus, pT4p2 might not originate from local Cu^R^ strains of *X. euvesicatoria* pv. *perforans*. However, we cannot exclude the possibility that other xanthomonads in other production areas in Taiwan, on other citrus or solanaceous cultivars which carry pLH3.1/pT4p2-like plasmids, could be found by increasing the number of Cu^R^ xanthomonad populations studied. A further survey of Cu^R^ xanthomonad populations on various hosts in combination with comparative genomic analysis will help to decipher the distribution and spread of Cu^R^ plasmids in xanthomonads.

Our whole genome sequencing data clarified that the *copLAB* cluster is located in the plasmid pT4p2 associated with heavy metal resistance. Horizontal transfer of copper resistance between bacteria occurs more frequently when the *cop* genes are located in the mobile plasmids (Behlau et al., 2012a). The *Tra* cluster responsible for the mobility of the plasmid was found in the Cu^R^ plasmid pT4p2. The data of conjugative transfer proved that pT4p2 is a mobile plasmid and able to horizontally transfer between different strains of *X. citri* subsp. *citri* (Supplementary Table 9). The Cu^S^ recipient cells became as resistant to copper as the donor cells while receiving *cop* genes *via* conjugation. Accordingly, the horizontal transfer of plasmid borne *cop* genes between citrus canker xanthomonads may potentially increase Cu^R^ xanthomonad populations to reduce the disease control efficacy of copper bactericides.

It is worth noting that the copper resistance gene clusters were located on the plasmid of *Xanthomonas* strains but were present in the chromosomes of *Stenotrophomonas, Pseudoxanthomonas, Lysobacter, Luteimonas* and *Thermomonas* (Figure 7). These Xanthomonadaceae microbes were either human pathogens (Crossman et al., 2008) or presented in the agricultural environment (Turrini et al., 2021) and some are known for carrying heavy metal resistance genes and mobile genetic elements. These environmental microbes could serve as a reservoir for the transfer of heavy metal resistance genes to microbes living in the surrounding environment.

We speculated that one copper resistance cluster in pT4p2 was inserted next to the arsenate cluster due to the Tn3-like transposon activity. Moreover, the additional copy of the arsenate and copper cluster might be the byproduct of conjugation where plasmids were transferred as a single-strand DNA during the conjugation and subsequently activated the bacterial SOS stress response. The recombination or mutagenesis frequencies of genomic fragments were then induced and increased the bacterial evolution rate (Rodriguez-Beltran et al., 2021). The high density of transposable elements around the heavy metal resistance clusters (Supplementary Figure 10) increased the probability that the duplicated fragment was inserted around the same location.

The whole genome sequences of the three strains in this study reflected the potential effect of agricultural practices or agroecosystems on diversification of citrus bacterial canker pathogens. A large sampling of microbes from the same environment over a long period of time would likely improve our understanding of how agricultural practices impact the microbial community.

## Conclusion

Complete genome sequencing of three *X. citri* subsp. *citri* pathotype A strains from two distant orchards and a comparison with the published genomes in this study clearly illustrated plasticity in chromosomes and plasmids. Our results revealed the evolution of pathogenicity factors and horizontal gene transfer events in the three strains. Type of agricultural management could be a potential trigger for evolution of pathotype A of *X. citri* subsp. *citri*. Surprisingly, conventional management might induce less deletion of CRISPR repeats in *X. citri* subsp. *citri* or increase the diversity of phages in the orchard. Thus, the *X. citri* subsp. *citri* strains under conventional management might have more CRIPSR repeats for immunity to phage infections compared with the strain from the orchard with minimal management. Moreover, the *cop* gene cluster together with the arsenate resistance gene cluster were only carried by the huge plasmid in the Cu^R^ strain of *X. citri* subsp. *citri*. Collectively, plasmids represented a hotspot for exchanging foreign genomic elements and accelerated the adaptation of *X. citri* subsp. *citri* to the agroecosystem.

## Data Availability Statement

The dataset generated for this study including sequencing reads, genome assemblies and genome annotation have been deposited at NCBI under BioProject PRJNA644481. The *X. citri* strains that support the findings of this study are available on request from C-JH.

## Author Contributions

C-JH, H-FN, and Y-CL conceived and designed the experiments. T-LW, P-XZ, and J-YO performed next-generation sequencing and bioinformatics analysis. C-JH, T-LW, and Y-CL interpreted the data. C-JH, T-LW, and Y-CL wrote the manuscript with input from all co-authors. All authors read and approved the final manuscript.

## Conflict of Interest

The authors declare that the research was conducted in the absence of any commercial or financial relationships that could be construed as a potential conflict of interest.

## Publisher’s Note

All claims expressed in this article are solely those of the authors and do not necessarily represent those of their affiliated organizations, or those of the publisher, the editors and the reviewers. Any product that may be evaluated in this article, or claim that may be made by its manufacturer, is not guaranteed or endorsed by the publisher.

## Footnotes

1
https://github.com/rambaut/figtree/releases

## Supplementary Material

Supplementary Figure 1 Highly conserved chromosome sequences of the three sequenced strains. Each collinearity block represents >5000 bp alignment and >99% sequence identity by BLASTN.

Supplementary Figure 2 Phylogenetic analysis based on whole genome comparison. The asterisks indicate copper-resistant strains.

Supplementary Figure 3 Shared and unique protein coding genes in three sequenced strains.

Supplementary Figure 4 Pangenome analysis of 79 *X. citri* genomes.

Supplementary Figure 5 Type II and III secretion systems. **(A)** Two operons of the type II secretion system. **(B)** Gene cluster of the type III secretion system.

Supplementary Figure 6 Pairwise comparison of plasmids. Upper panel: copper resistance plasmids. Lower panel: pathogenicity related plasmids. Blue line: syntenic region in the same direction. Green: syntenic region in the reverse direction. Orange: secondary alignment. a. plasmids sequenced in this study.

Supplementary Figure 7 Estimated plasmid copy number based on the Nanopore sequencing coverage.

Supplementary Figure 8 Multiple plasmid alignment of pXAC33, pXAC64 and non-CuR plasmids sequenced in this study. **(A)** Mauve alignment including pXAC33, pxAC64 and plasmids in this study. **(B)** Mauve alignment of plasmids in this study. Syntenic regions share the same color.

Supplementary Figure 9 Genome assembly confirmation of the copper-resistance plasmid pT4p2. **(A)** Genome structure of the 40 kbp inverted repeat. **(B)** PCR product of the PF1-CF1 primer pair and **(C)** PCR product of the PF1-CR3 primer pair.

Supplementary Figure 10 Inverted duplication of metal-resistance clusters between pT4p2 and pLH3.1. Orange box (Cop): location of copper resistance gene clusters. Pink box (Ars): arsenate-resistant gene clusters. Tns: transposase.

Supplementary Figure 11 Comparison of plasmids containing copper resistance genes in pT4p2, pCur and pLH201.1. Circles from outermost to innermost represent: (1) position and genes, the grid is 10 kbp [pink, pT4p2 of strain T4; yellow, pLH201.1 (NZ_CP018859.1) of strain LH201; white, pCuR (NZ_CP023286.1) of strain 03-1638-1-1]; (2) GC content with 2000-bp sliding window and 200-bp step; (3) regions with similar gene content and nucleotide sequence identity >90%, and alignment length >1 kb between plasmids.

Supplementary Figure 12 Multiple sequence alignment of csd1 and cas8c genes. Black box, around 530 nt, indicates the AG frameshift.

Supplementary Table 1 Summary of assembly and annotation statistics.

Supplementary Table 2 Strains used for pan-genome analysis.

Supplementary Table 3 Pairwise nucleotide sequences identified by fastANI.

Supplementary Table 4 GO enrichment of strain specific genes.

Supplementary Table 5 Gene clsters of the type II and type III secretion systems.

Supplementary Table 6 TALEs and repeat number of RVDs in plasmids.

Supplementary Table 7 Strains used for copper and arsenate resistance gene cluster alignment.

Supplementary Table 8 Strains containing the unique spacer repeat between the copper and arsenate resistance gene clusters.

Supplementary Table 9 Conjugation frequency of plasmid-borne copper resistance genes transferred between different *Xanthomonas citri* subsp. *citri* strains *in vitro*.
