# Supplementary material for: Comparative Genomic Analysis Uncovered Evolution of Pathogenicity Factors, Horizontal Gene Transfer Events, and Heavy Metal Resistance Traits in Citrus Canker Bacterium Xanthomonas citri subsp. citri
Source: Front Microbiol. 2021 Sep 7;12:731711. doi: 10.3389/fmicb.2021.731711 (PMC8453159; doi:10.3389/fmicb.2021.731711)
Supplement: Supplementary Figure 1 — Highly conserved chromosome sequences of the three sequenced strains. Each collinearity block represents >5000 bp alignment and >99% sequence identity by BLASTN. [file Presentation_1.pdf]

Comparative genomic analysis uncovered evolution of pathogenicity factors, horizontal gene transfer events and heavy metal resistance traits in citrus canker bacterium *Xanthomonas citri* subsp. *citri*

Chien-Jui Huan, Ting-Li W, Po-Xing Zheng, Jheng-Yang Ou, Hui-Fang Ni, Yao-Cheng Lin

Front. Microbiol. 12:731711.

doi: 10.3389/fmicb.2021.731711

Supplementary Material

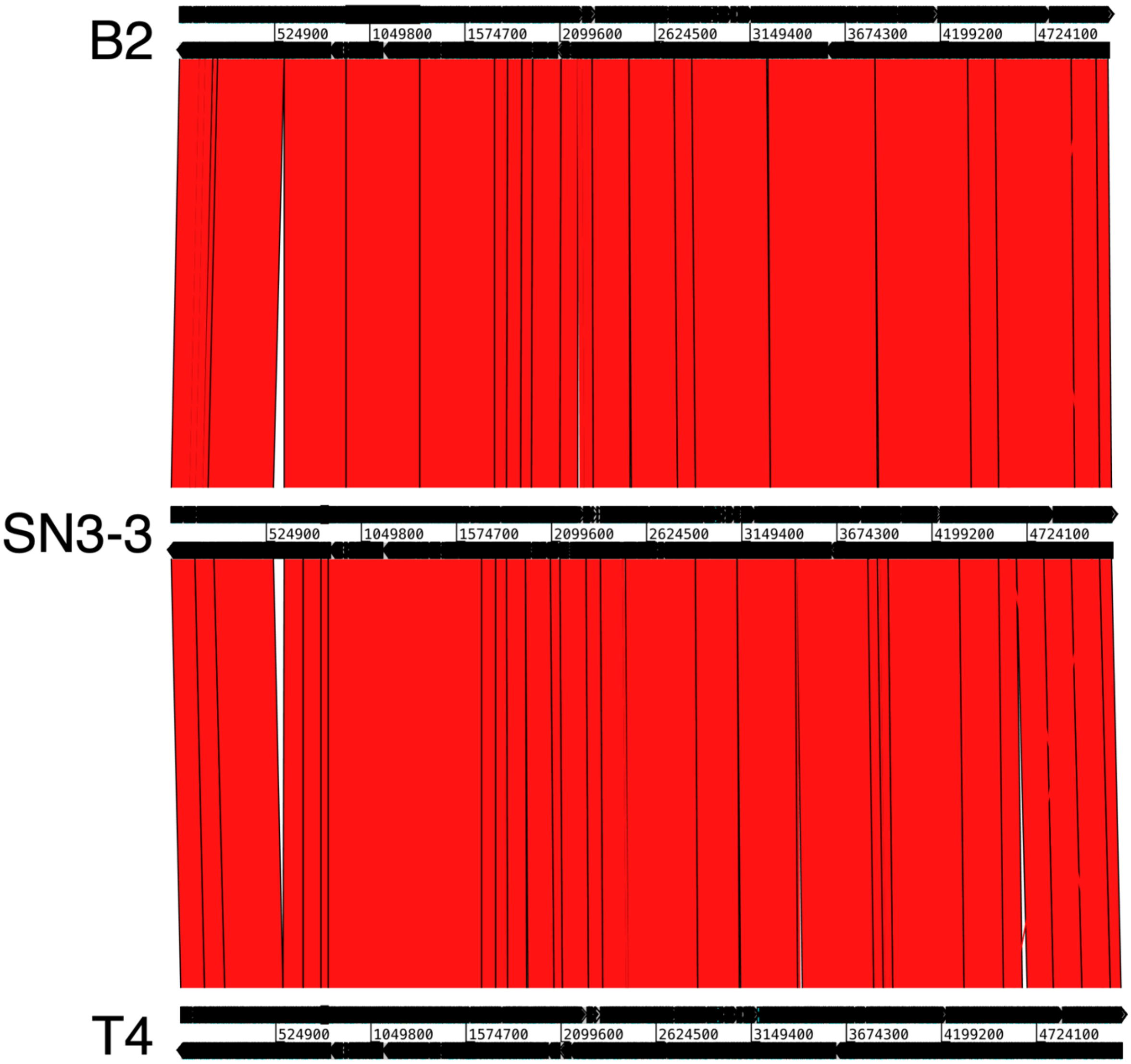

Supplementary Figure 1. Highly conserved chromosome sequences of the three sequenced strains. Each collinearity block represents >5,000 bp alignment and >99% sequence identity by BLASTN.

doi: 10.3389/fmicb.2021.731711

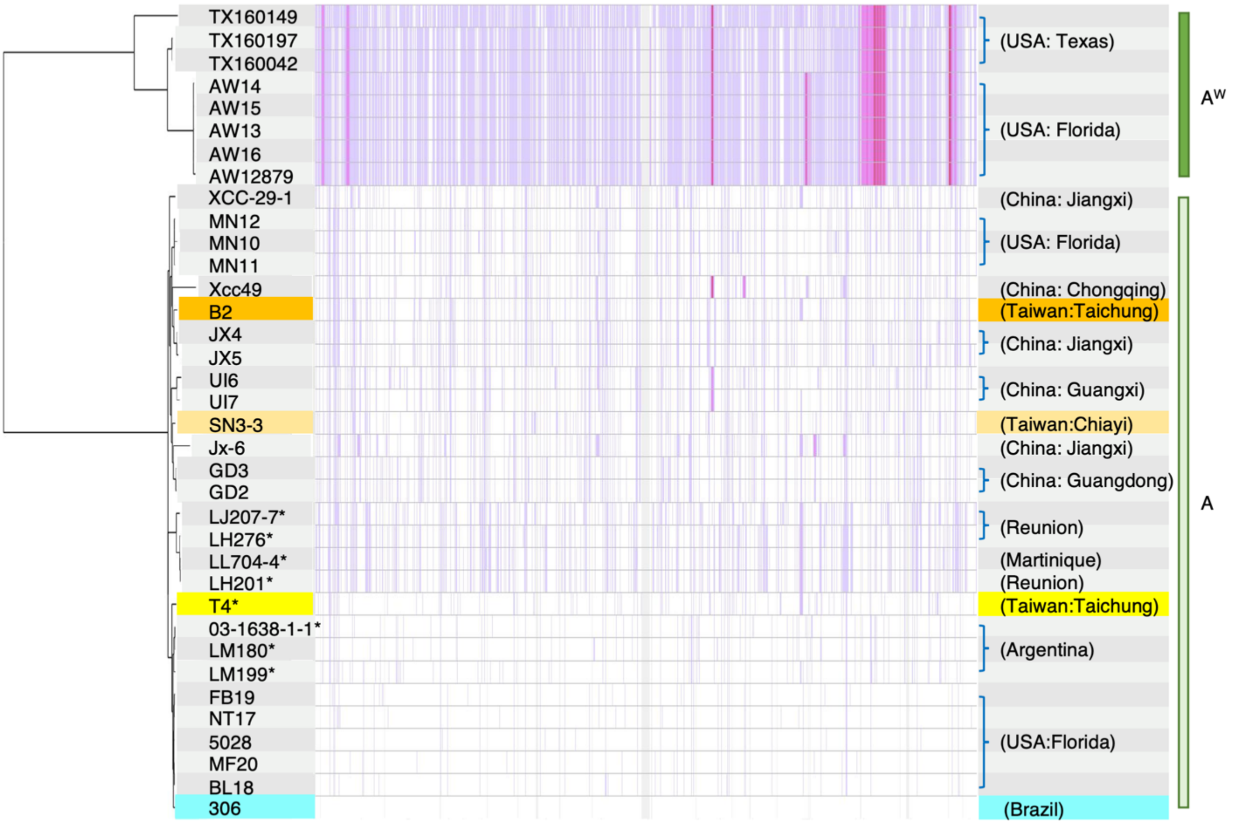

Supplementary Figure 2. Phylogenetic analysis based on whole genome comparison. The asterisks indicate copper-resistant strains.

 doi: 10.3389/fmicb.2021.731711

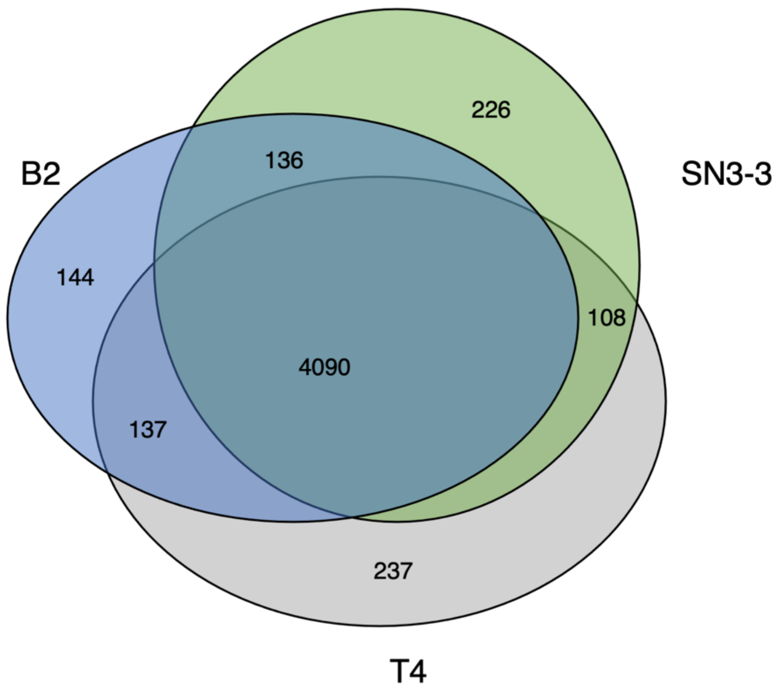

Supplementary Figure 3. Shared and unique protein coding genes in three sequenced strains.

doi: 10.3389/fmicb.2021.731711

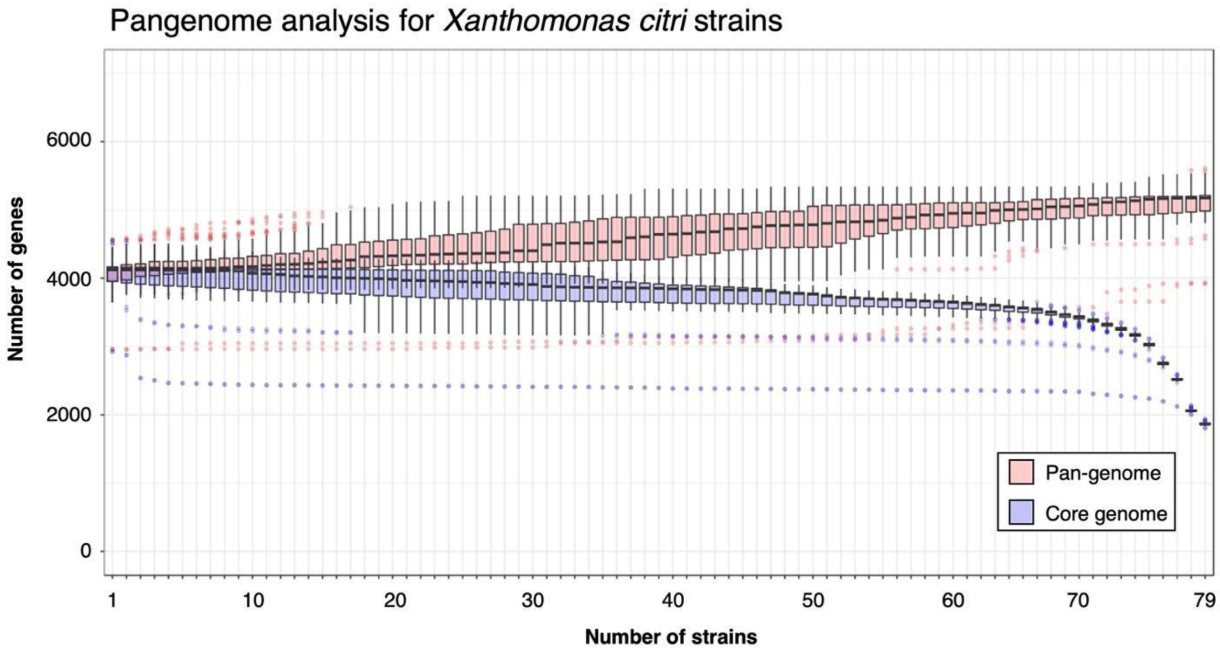

Supplementary Figure 4. Pangenome analysis of 79 Xanthomonas genomes.

 doi: 10.3389/fmicb.2021.731711

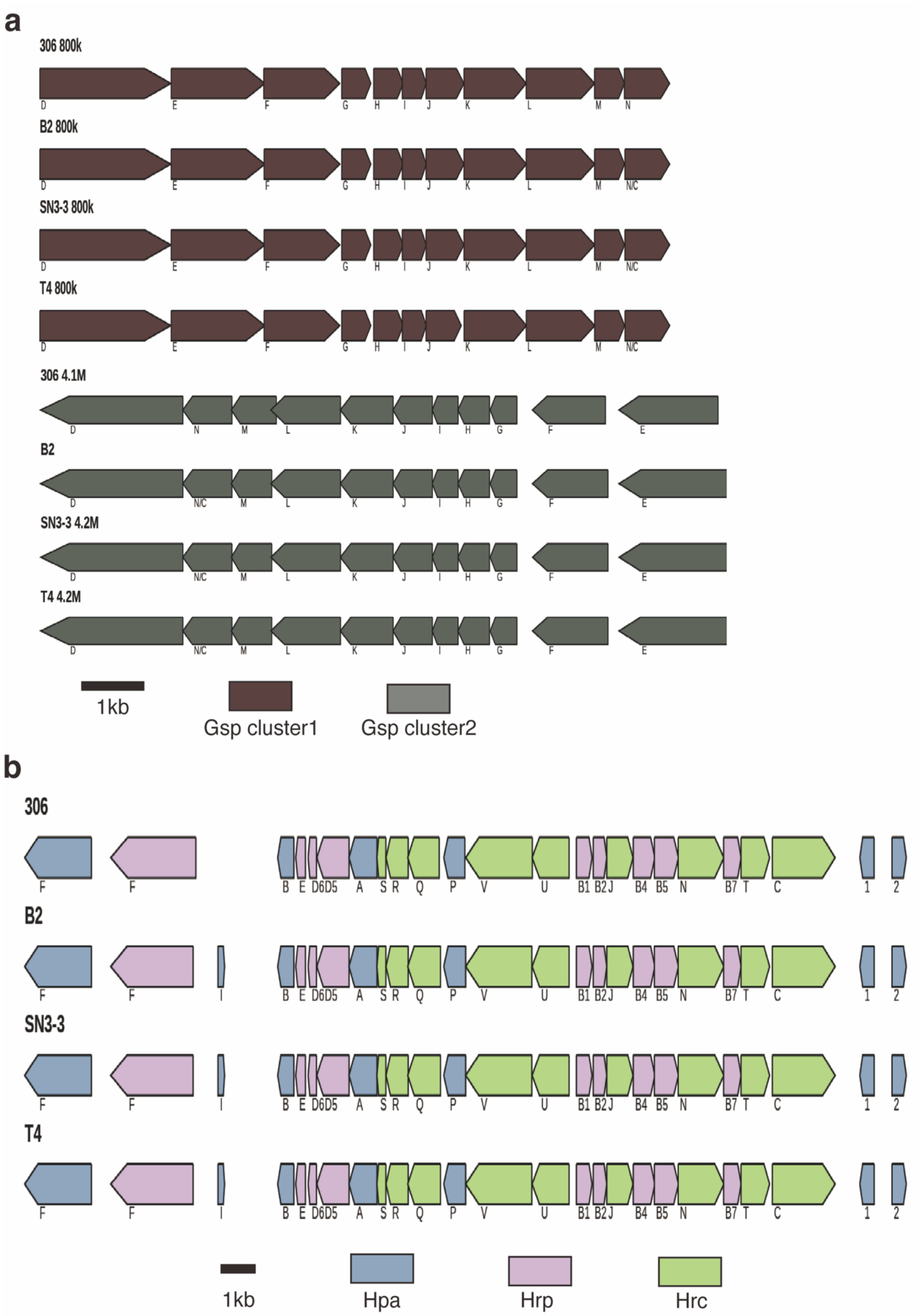

Supplementary Figure 5. Type II and III secretion systems. (A) Two operons of the type II secretion system. (B) Gene cluster of the type III secretion system

 doi: 10.3389/fmicb.2021.731711

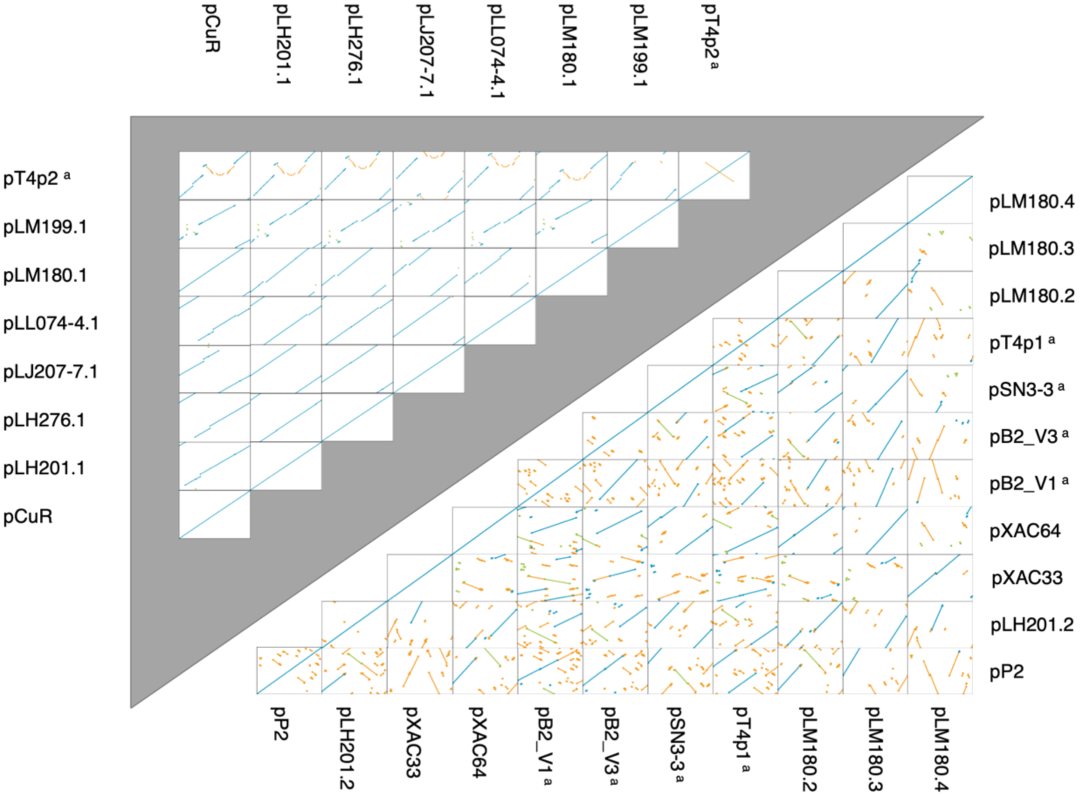

Supplementary Figure 6. Pairwise comparison of plasmids. Upper panel: copper resistance plasmids. Lower panel: pathogenicity related plasmids. Blue line: syntenic region in the same direction. Green: syntenic region in the reverse direction. Orange: secondary alignment. a. plasmids sequenced in this study.

 doi: 10.3389/fmicb.2021.731711

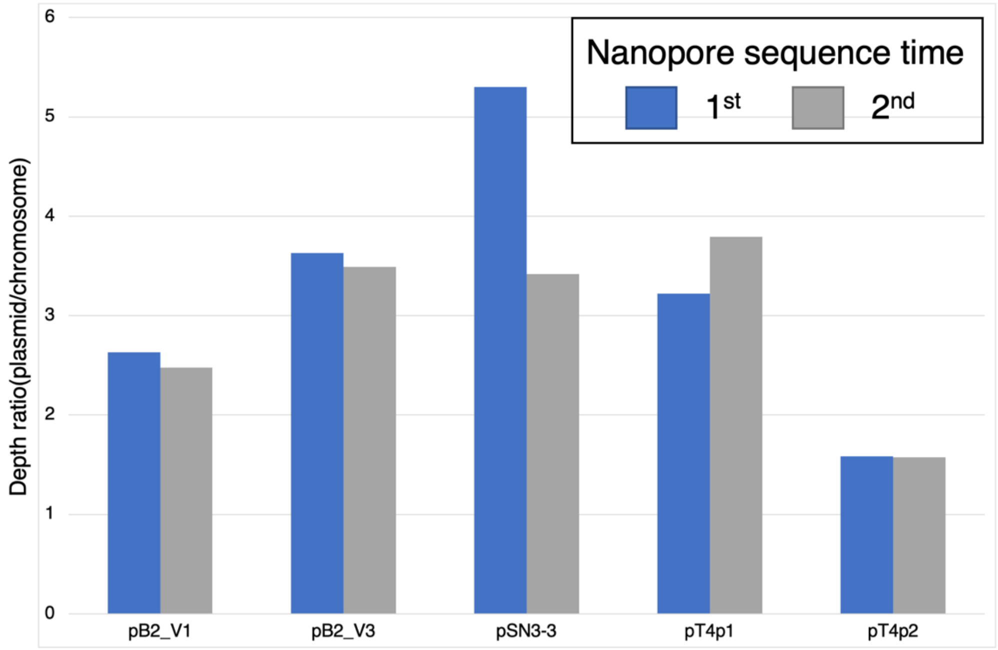

Supplementary Figure 7. Estimated plasmid copy number based on the Nanopore sequencing coverage.

 doi: 10.3389/fmicb.2021.731711

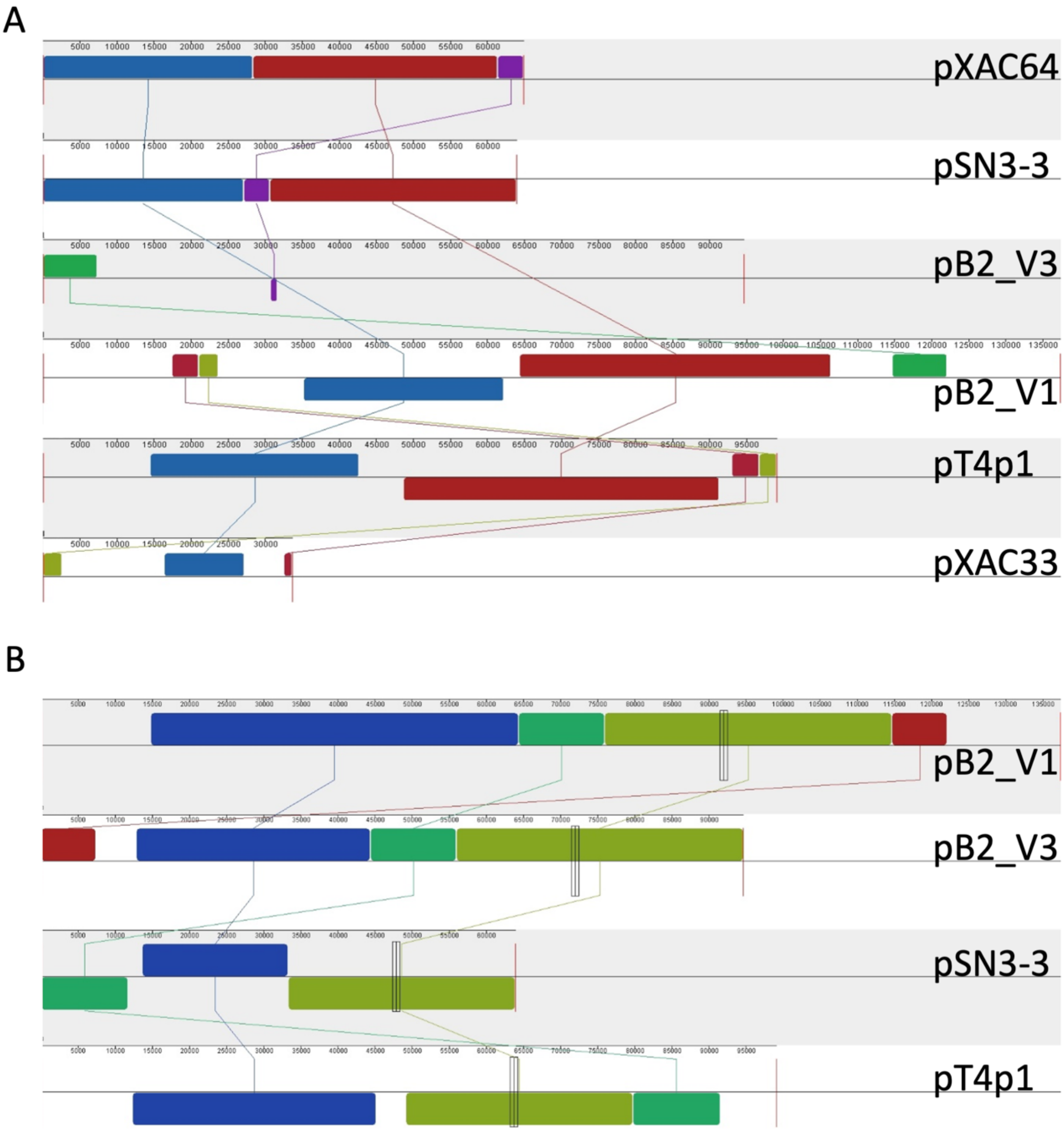

Supplementary Figure 8. Multiple plasmid alignment of pXAC33, pXAC64 and non-CuR plasmids sequenced in this study. (A) Mauve alignment including pXAC33, pxAC64 and plasmids in this study. (B) Mauve alignment of plasmids in this study. Syntenic regions share the same color.

doi: 10.3389/fmicb.2021.731711

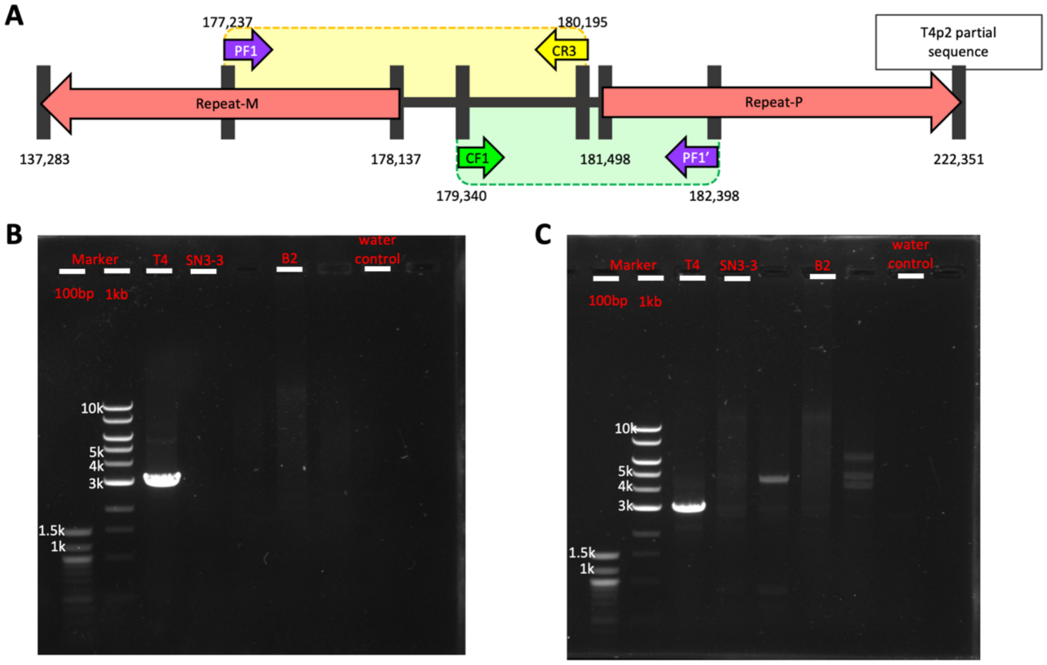

Supplementary Figure 9. Genome assembly confirmation of the copper-resistance plasmid pT4p2. (A) Genome structure of the 40 kbp inverted repeat. (B) PCR product of the PF1-CF1 primer pair and (C) PCR product of the PF1-CR3 primer pair.

doi: 10.3389/fmicb.2021.731711

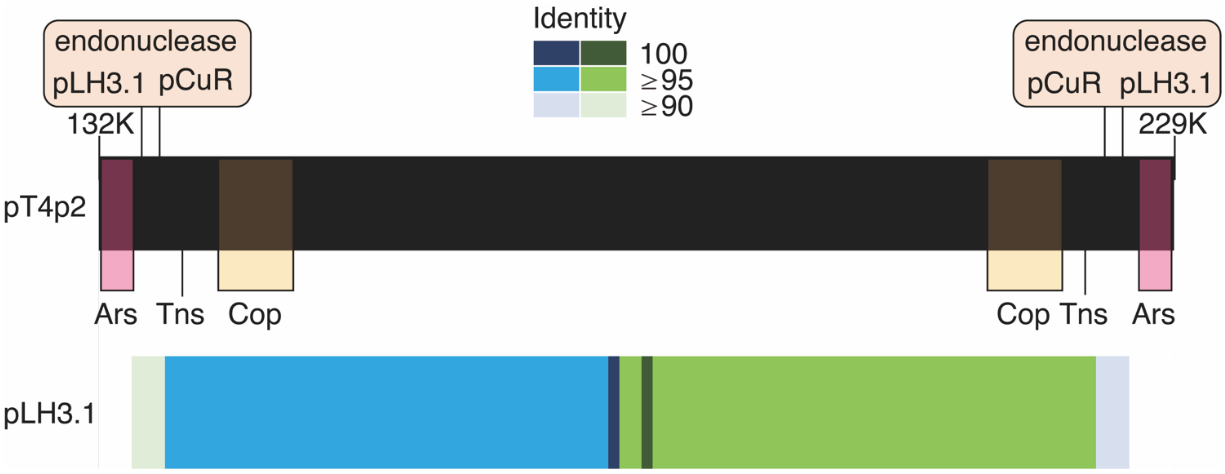

Supplementary Figure 10. Inverted duplication of metal-resistance clusters between pT4p2 and pLH3.1. Orange box (Cop): location of copper resistance gene clusters. Pink box (Ars): arsenate-resistant gene clusters. Tns: transposase.

 doi: 10.3389/fmicb.2021.731711

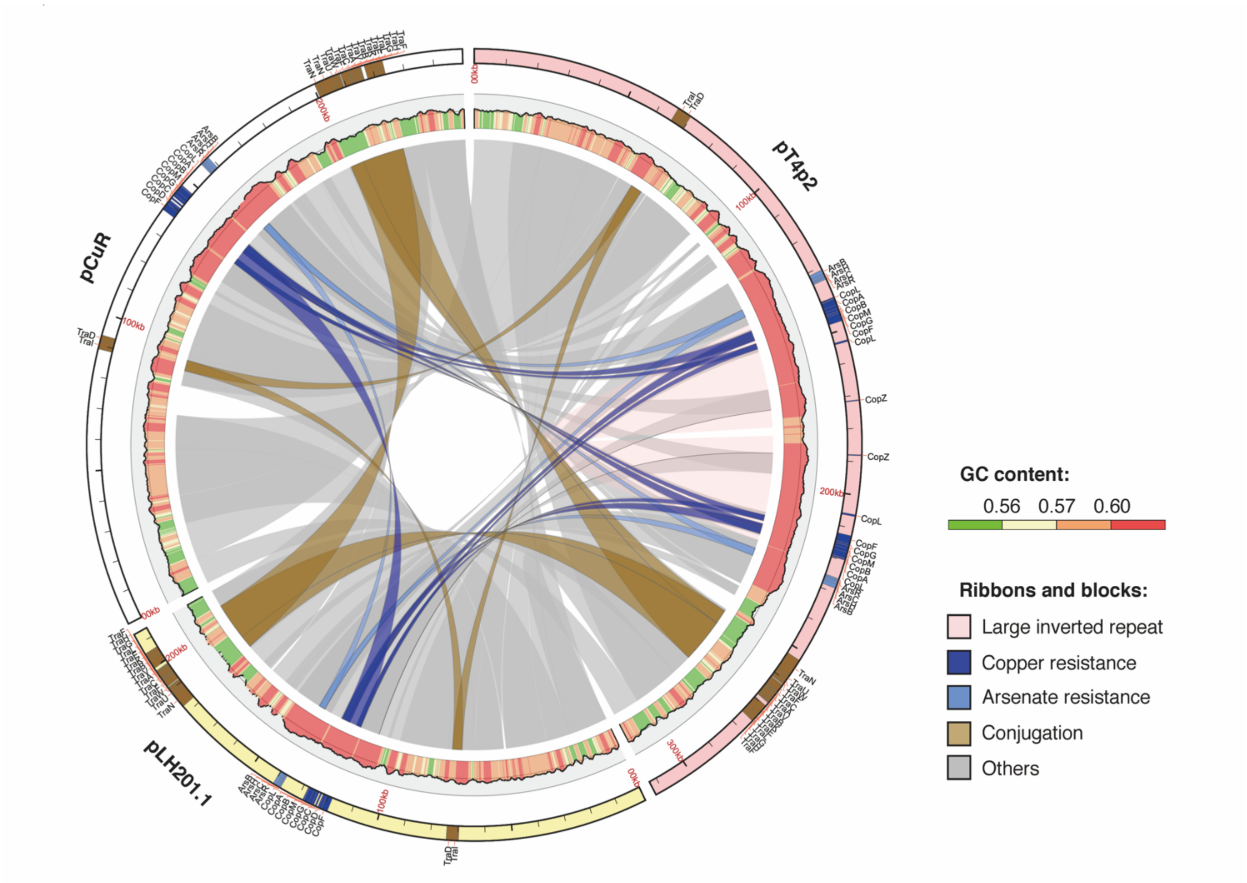

Supplementary Figure 11. Comparison of plasmids containing copper resistance genes in pT4p2, pCur and pLH201.1. Circles from outermost to innermost represent: (1) position and genes, the grid is 10 kbp [pink, pT4p2 of strain T4; yellow, pLH201.1 (NZ_CP018859.1) of strain LH201; white, pCuR (NZ_CP023286.1) of strain 03-1638-1-1; (2) GC content with 2000-bp sliding window and 200-bp step; (3) regions with similar gene content and nucleotide sequence identity > 90%, and alignment length > 1kb between plasmids.

doi: 10.3389/fmicb.2021.731711

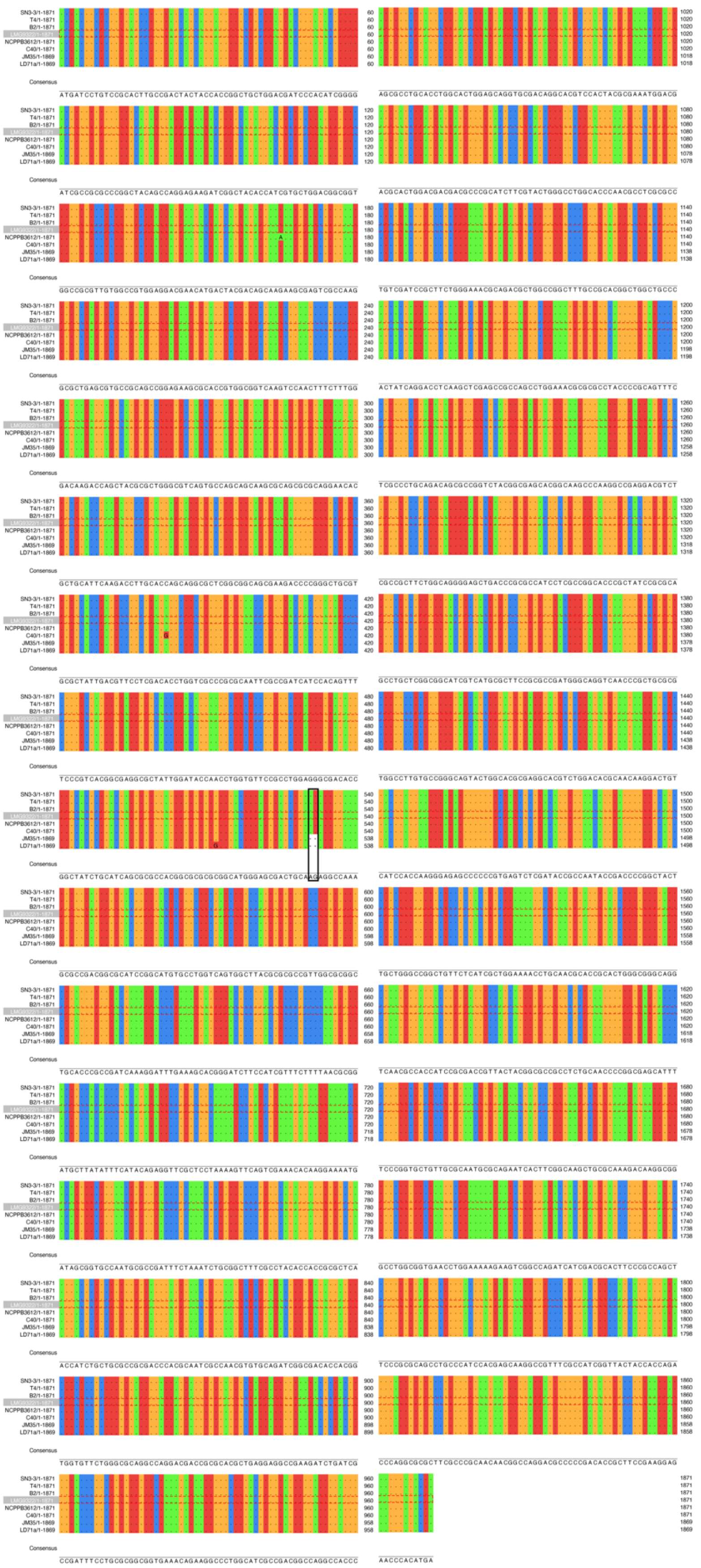

Supplementary Figure 12. Multiple sequence alignment of csd1 and cas8c genes. Black box, around 530 nt, indicates the AG frameshift.

doi: 10.3389/fmicb.2021.731711
